# Supplementary material for: Novel Sulfonylurea Derivatives as Potential Antimicrobial Agents: Chemical Synthesis, Biological Evaluation, and Computational Study
Source: Antibiotics (Basel). 2023 Feb 3;12(2):323. doi: 10.3390/antibiotics12020323 (PMC9951967; doi:10.3390/antibiotics12020323)
Supplement: Supplementary file 1 [file antibiotics-12-00323-s001.zip › antibiotics-2171242-supplementary-1.pdf]

Article

Novel Sulfonylurea Derivatives as Potential Antimicrobial Agents: Chemical Synthesis, Biological Evaluation, and Computational Study

Fan-Fei Meng [1], Ming-Hao Shang [1], Wei Wei [1,†], Zhen-Wu Yu [1], Jun-Lian Liu [2], Zheng-Ming Li [1,‡], Zhong-Wen Wang [1], Jian-Guo Wang[1,*] and Huan-Qin Dai [3,4,*]

1 State-Key Laboratory and Research Institute of Elemento-Organic Chemistry, Frontiers Science Center for New Organic Matter, College of Chemistry, Nankai University, Tianjin 300071, China
2 Scientific Research Training Center for Chinese Astronauts, Beijing, 100094, China
3 State Key Laboratory of Mycology, Institute of Microbiology, Chinese Academy of Sciences, Beijing 100101, China
4 Savaid Medical School, University of Chinese Academy of Sciences, Beijing, 100049, China
† Present address, WuXi AppTec (Tianjin) Co., Ltd., Tianjin 300071, China.
‡ Zheng-Ming Li is deceased.
* Correspondence: nkwjg@nankai.edu.cn (J.-G. W.); orcid.org/0000-0001-7577-1502; daihq@im.ac.cn (H.-Q. D.); orcid.org/0000-0002-8108-815X.

Molecular structures of the ten commercial sulfonylurea herbicides…………………………...2
The structures and characterization data of intermediates 4a-4u……………………………….3
The structures and characterization data of target compounds 9a-9u………………………….7

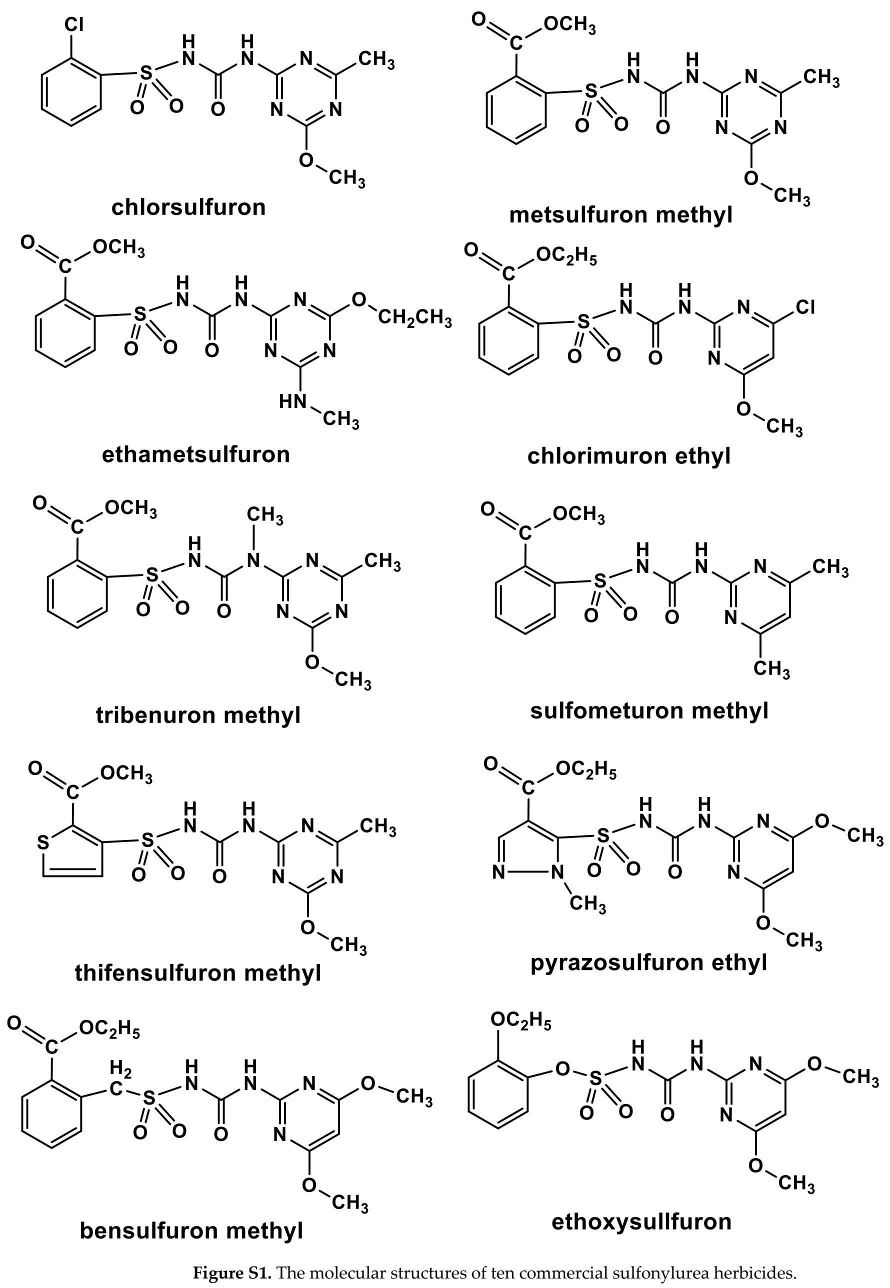

Figure S1. The molecular structures of ten commercial sulfonylurea herbicides.

The structures and characterization data of intermediates 4a-4u

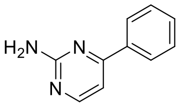

4a

4-phenylpyrimidin-2-amine (4a): White solid; Yield: 78.5%; m.p.: 158-159 ^{o}C; ^{1}H NMR (400 MHz, DMSO-d_6) δ 8.32 (d, *J* = 5.16 Hz, 1H, pyrim-H), 8.12 – 8.04 (m, 2H, Ph-H), 7.54 – 7.48 (m, 3H, Ph-H), 7.13 (d, *J* = 5.17 Hz, 1H, pyrim-H), 6.71 (s, 2H, NH_2).

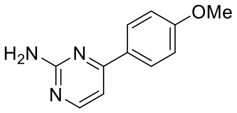

4b

4-(4-methoxyphenyl)pyrimidin-2-amine (4b): White solid; Yield: 81.6%; m.p.: 176-177 ^{o}C; ^{1}H NMR (400 MHz, DMSO-d_6) δ 8.25 (d, *J* = 5.25 Hz, 1H, pyrim-H), 8.05 (d, *J* = 8.88 Hz, 2H, Ph-H), 7.07 (d, *J* = 5.30 Hz, 1H, pyrim-H), 7.04 (d, *J* = 8.88 Hz, 2H, Ph-H), 6.60 (s, 2H, NH_2), 3.82 (s, 3H, OCH_3).

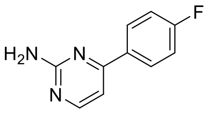

4c

4-(4-fluorophenyl)pyrimidin-2-amine (4c): White solid; Yield: 77.7%; m.p.: 162-163 ^{o}C; ^{1}H NMR (400 MHz, DMSO-d_6) δ 8.32 (d, *J* = 5.23 Hz, 1H, pyrim-H), 8.20 – 8.10 (m, 2H, Ph-H), 7.39 – 7.29 (m, 2H, Ph-H), 7.13 (d, *J* = 5.20 Hz, 1H, pyrim-H), 6.73 (s, 2H, NH_2).

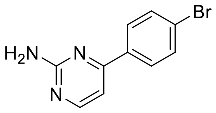

4d

4-(4-bromophenyl)pyrimidin-2-amine (4d): White solid; Yield: 86.9%; m.p.: 181-182 ^{o}C; ^{1}H NMR (400 MHz, DMSO-d_6) δ 8.35 (d, *J* = 5.18 Hz, 1H, pyrim-H), 8.04 (d, *J* = 8.58 Hz, 2H, Ph-H), 7.71 (d, *J* = 8.56 Hz, 2H, Ph-H), 7.15 (d, *J* = 5.19 Hz, 1H, pyrim-H), 6.78 (s, 2H, NH_2).

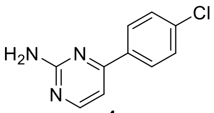

4e

4-(4-chlorophenyl)pyrimidin-2-amine (4e): White solid; Yield: 91.1%; m.p.: 170-171 ^{o}C; ^{1}H NMR (400 MHz, DMSO-d_6) δ 8.34 (d, *J* = 5.13 Hz, 1H, pyrim-H), 8.11 (d, *J* = 8.47 Hz, 2H, Ph-H), 7.57 (d, *J* = 8.47 Hz, 2H, Ph-H), 7.15 (d, *J* = 5.15 Hz, 1H, pyrim-H), 6.77 (s, 2H, NH_2).

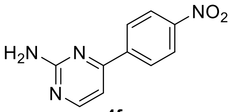

4f

4-(4-nitrophenyl)pyrimidin-2-amine (4f): Yellow solid; Yield: 69.4%; m.p.: 169-170 °C; ^{1}H NMR (400 MHz, DMSO-d_6) δ 8.42 (d, *J* = 5.08 Hz, 1H, pyrim-H), 8.39 – 8.29 (m, 4H, Ph-H), 7.27 (d, *J* = 5.14 Hz, 1H, pyrim-H), 6.89 (s, 2H, NH_2).

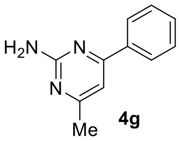

4g

4-methyl-6-phenylpyrimidin-2-amine (4g): White solid; Yield: 78.5%; m.p.: 166-167 °C; ^{1}H NMR(400 MHz, DMSO-d_6): *δ* 8.10 – 8.02 (m, 2H, Ph-H), 7.52 – 7.46 (m, 3H, Ph-H), 7.05 (s, 1H, pyrim-H), 6.57 (s, 2H, NH_2), 2.31 (s, 3H, pyrim-CH_3).

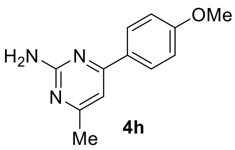

4h

4-(4-methoxyphenyl)-6-methylpyrimidin-2-amine (4h): White solid; Yield: 86.1%; m.p.: 181-182 °C; ^{1}H NMR(400 MHz, DMSO-d_6): *δ* 8.03 (d, *J* = 8.43 Hz, 2H, Ph-H), 7.03 (d, *J* = 8.49 Hz, 2H, Ph-H), 6.99 (s, 1H, pyrim-H), 6.50 (s, 2H, NH_2), 3.82 (s, 3H, Ph-OCH_3), 2.28 (s, 3H, pyrim-CH_3).

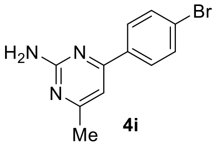

4i

4-(4-bromophenyl)-6-methylpyrimidin-2-amine (4i): White solid; Yield: 78.3%; m.p.: 163-164 °C; ^{1}H NMR(400 MHz, DMSO-d_6): *δ* 8.01 (d, *J* = 7.78 Hz, 2H, Ph-H), 7.70 (d, *J* = 7.78 Hz, 2H, Ph-H), 7.07 (s, 1H, pyrim-H), 6.63 (s, 2H, NH_2), 2.30 (s, 3H, pyrim-CH_3).

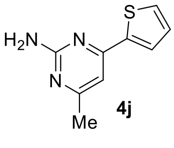

4j

4-methyl-6-(thiophen-2-yl)pyrimidin-2-amine (4j): White solid; Yield: 60.0%; m.p.: 149-150 °C; ^{1}H NMR (400 MHz, DMSO-d_6) δ 7.85 (dd, *J* = 3.77, 1.17 Hz, 1H, thienyl-H), 7.70 (dd, *J* = 5.04, 1.13 Hz, 1H, thienyl-H), 7.18 (dd, *J* = 5.02, 3.69 Hz, 1H, thienyl-H), 6.99 (s, 1H, pyrim-H), 6.59 (s, 2H, NH_2), 2.27 (s, 3H, pyrim-CH_3).

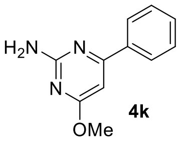

4k

4-methoxy-6-phenylpyrimidin-2-amine (4k): White solid; Yield: 59.4%; m.p.: 155-156 °C; ^{1}H NMR(400 MHz, DMSO-d_6): δ 8.08 – 7.97 (m, 2H, Ph-H), 7.52 – 7.39 (m, 3H, Ph-H), 6.65 (s, 2H, NH_2), 6.56 (s, 1H, pyrim-H), 3.86 (s, 3H, pyrim-OCH_3).

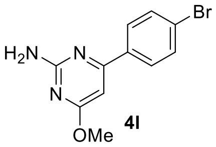

4l

4-(4-bromophenyl)-6-methoxypyrimidin-2-amine (4l): White solid; Yield: 93.5%; m.p.: 157-158 °C; ^{1}H NMR (400 MHz, DMSO-d_6) δ 7.99 (d, *J* = 8.63 Hz, 2H, Ph-H), 7.67 (d, *J* = 8.56 Hz, 2H, Ph-H), 6.72 (s, 2H, NH_2), 6.59 (s, 1H, pyrim-H), 3.86 (s, 3H, OCH_3).

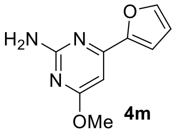

4m

4-(furan-2-yl)-6-methoxypyrimidin-2-amine (4m): White solid; Yield: 79.4%; m.p.: 123-124 °C; ^{1}H NMR (400 MHz, DMSO-d_6) δ 7.84 (d, *J* = 3.62 Hz, 1H, furyl-H), 7.67 (d, *J* = 4.91 Hz, 1H, furyl-H), 7.16 (t, *J* = 4.27 Hz, 1H, furyl-H), 6.67 (s, 2H, NH_2), 6.54 (s, 1H, pyrim-H), 3.84 (d, *J* = 1.05 Hz, 3H, OCH_3).

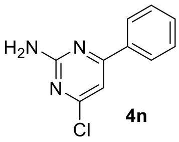

4n

4-chloro-6-phenylpyrimidin-2-amine (4n): Yellow solid; Yield: 86.6%; m.p.: 145-146 °C; ^{1}H NMR(400 MHz, DMSO-d_6): δ 8.12 – 8.06 (m, 2H, Ph-H), 7.54 – 7.47 (m, 3H, Ph-H), 7.25 (s, 1H, pyrim-H), 7.20 (s, 2H, NH_2).

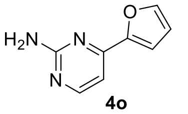

4o

4-(furan-2-yl)pyrimidin-2-amine (4o): White solid; Yield: 88.3%; m.p.: 164-166 °C; ^{1}H NMR(400 MHz, DMSO-d_6): δ 8.27 (d, *J* = 5.09 Hz, 1H, pyrim-H), 7.88 (s, 1H, furyl-H), 7.23 – 7.11 (m, 1H, furyl-H), 6.87 (d, *J* = 5.10 Hz, 1H, pyrim-H), 6.73 – 6.62 (m, 3H, furyl-H & NH_2).

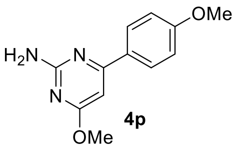

4p

4-methoxy-6-(4-methoxyphenyl)pyrimidin-2-amine (4p): White solid; Yield: 80.3%; m.p.: 126-127 °C; ^{1}H NMR(400 MHz, DMSO-d_6): δ 8.01 (d, J = 8.64 Hz, 2H, Ph-H), 7.01 (d, J = 8.70 Hz, 2H, Ph-H), 6.58 (s, 2H, NH_2), 6.50 (s, 1H, pyrim-H), 3.84 (s, 3H, pyrim-OCH_3), 3.81 (s, 3H, Ph-OCH_3).

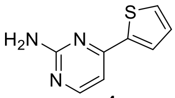

4q

4-(thiophen-2-yl)pyrimidin-2-amine (4q): Yellow solid; Yield: 90.2%; m.p.: 162-163 °C; ^{1}H NMR(400 MHz, DMSO-d_6): δ 8.28 (d, J = 5.04 Hz, 1H, pyrim-H), 7.92 (d, J = 7.58 Hz, 1H, thienyl-H), 7.85 – 7.65 (m, 1H, thienyl-H), 7.29 – 7.03 (m, 2H, thienyl-H & pyrim-H), 6.69 (s, 2H, NH_2).

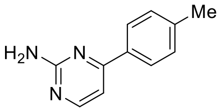

4r

4-(*p*-tolyl)pyrimidin-2-amine (4r): White solid; Yield: 70.2%; m.p.: 189-190 °C; ^{1}H NMR(400 MHz, DMSO-d_6): δ 8.28 (d, J = 5.16 Hz, 1H, pyrim-H), 7.98 (d, J = 7.82 Hz, 2H, Ph-H), 7.31 (d, J = 7.85 Hz, 2H, Ph-H), 7.10 (d, J = 5.19 Hz, 1H, pyrim-H), 6.64 (s, 2H, NH_2), 2.37 (s, 3H, Ph-CH_3).

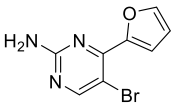

4s

5-bromo-4-(furan-2-yl)pyrimidin-2-amine (4s): White solid; Yield: 80.6%; m.p.: 204-206 °C; ^{1}H NMR(400 MHz, DMSO-d_6): δ 8.41 (s, 1H, pyrim-H), 7.96 (dd, J = 1.71, 0.66 Hz, 1H, furyl-H), 7.50 (dd, J = 3.58, 0.64 Hz, 1H, furyl-H), 6.94 (s, 2H, NH_2), 6.73 (dd, J = 3.52, 1.72 Hz, 1H, furyl-H)

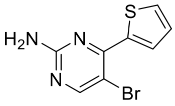

4t

5-bromo-4-(thiophen-2-yl)pyrimidin-2-amine (4t): White solid; Yield: 85.5%; m.p.: 156-158 °C; ^{1}H NMR(400 MHz, DMSO-d_6): δ 8.53 (s, 1H, pyrim-H), 8.32 (d, J = 3.50 Hz, 1H, thienyl-H), 7.94 (d, J = 4.79 Hz, 1H, thienyl-H), 7.30–7.23 (m, 1H, thienyl-H), 6.17 (s, 2H, NH_2).

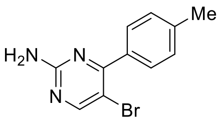

4u

5-bromo-4-(4-methoxyphenyl)pyrimidin-2-amine (4u): White solid; Yield: 86.2%; m.p.: 155-156 °C; ^{1}H NMR (400 MHz, DMSO-d_6) δ 8.45 (s, 1H, pyrim-H), 8.35 (d, *J* = 8.58 Hz, 2H, Ph-H), 7.24 (d, *J* = 8.56 Hz, 2H, Ph-H), 6.75 (s, 2H, NH_2), 2.40 (s, 3H, Ph-CH_3).

The structures and characterization data of target compounds 9a-9u

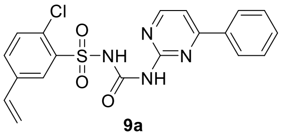

9a

2-chloro-*N*-((4-phenylpyrimidin-2-yl)carbamoyl)-5-vinylbenzenesulfonamide (9a): White solid; Yield: 76.5%; m. p.: 235-237 °C; HRMS for $C_{19}H_{15}ClN_4O_3S$: calcd 415.0626 [M+H]$^+$, found 415.0628; ^{1}H NMR (400 MHz, DMSO) δ 13.45 (s, 1H, SO_2N**H**CONH), 10.90 (s, 1H, SO_2NHCON**H**)), 8.80 (d, *J* = 5.2 Hz, 1H, pyrim-H), 8.21 (s, 1H, Ph-H), 8.19 (s, 2H, Ph-H), 7.89 (d, *J* = 8.2 Hz, 1H, Ph-H), 7.84 (d, *J* = 5.4 Hz, 1H, pyrim-H), 7.68 (d, *J* = 8.3 Hz, 1H, Ph-H), 7.65 – 7.55 (m, 3H, Ph-H), 6.91 (dd, *J* = 17.7, 10.9 Hz, 1H, ArC**H**=CH_2), 6.02 (d, *J* = 17.7 Hz, 1H, ArCH=C**H**$_2$), 5.48 (d, *J* = 10.9 Hz, 1H, ArCH=C**H**$_2$); ^{13}C NMR (101 MHz, DMF) δ 164.85, 159.43, 157.94, 149.26, 137.49, 137.09, 135.58, 134.75, 132.52, 132.38, 132.26, 130.33, 130.12, 129.48, 127.62, 117.29, 112.04.

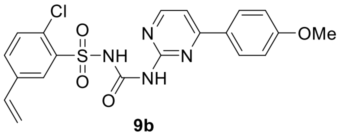

9b

2-chloro-*N*-((4-(4-methoxyphenyl)pyrimidin-2-yl)carbamoyl)-5-vinylbenzenesulfonamide (9b):White solid; Yield: 68.8%; m. p.:216-218 °C; HRMS for $C_{20}H_{17}ClN_4O_4S$: calcd 445.0732 [M+H]$^+$, found 445.0732; ^{1}H NMR (400 MHz, DMSO) δ 13.62 (s, 1H, SO_2N**H**CONH), 10.87 (s, 1H, SO_2NHCON**H**), 8.71 (d, *J* = 4.7 Hz, 1H, pyrim-H), 8.20 (s, 2H, Ph-H), 8.18 (s, 1H, Ph-H), 7.89 (d, *J* = 8.1 Hz, 1H, Ph-H), 7.77 (d, *J* = 5.4 Hz, 1H, pyrim-H), 7.67 (d, *J* = 8.2 Hz, 1H, Ph-H), 7.12 (d, *J* = 8.0 Hz, 2H, Ph-H), 6.91 (dd, *J* = 17.5, 11.0 Hz, 1H, ArC**H**=CH_2), 6.02 (d, *J* = 17.7 Hz, 1H, ArCH=C**H**$_2$), 5.48 (d, *J* = 11.0 Hz, 1H, ArCH=C**H**$_2$), 3.87 (s, 3H, OCH_3); ^{13}C NMR (101 MHz, DMSO) δ 163.39, 162.23, 158.11, 156.54, 148.60, 136.48, 133.98, 131.85, 131.59, 129.47, 129.04, 128.83, 126.67, 117.28, 114.30, 113.75, 110.56, 55.27.

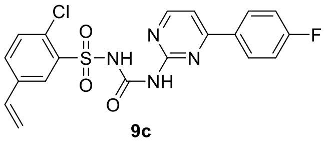

9c

2-chloro-*N*-((4-(4-fluorophenyl)pyrimidin-2-yl)carbamoyl)-5-vinylbenzenesulfonamide (9c):White solid; Yield: 73.5%; m. p.:230-231 °C; HRMS for $C_{19}H_{14}ClFN_4O_3S$: calcd 433.0532 [M+H]$^+$, found 433.0533; ^{1}H NMR (400 MHz, DMSO) δ 13.34 (s, 1H,

SO_2N**H**CONH), 10.88 (s, 1H, SO_2NHCON**H**), 8.79 (d, *J* = 5.2 Hz, 1H, pyrim-H), 8.27 (dd, *J* = 8.1, 5.6 Hz, 2H, Ph-H), 8.19 (s, 1H, Ph-H), 7.89 (d, *J* = 8.2 Hz, 1H, Ph-H), 7.84 (d, *J* = 5.4 Hz, 1H, pyrim-H), 7.68 (d, *J* = 8.3 Hz, 1H, Ph-H), 7.44 (t, *J* = 8.6 Hz, 2H, Ph-H), 6.91 (dd, *J* = 17.6, 11.0 Hz, 1H, ArC**H**=CH_2), 6.02 (d, *J* = 17.7 Hz, 1H, ArCH=C**H**$_2$), 5.48 (d, *J* = 11.0 Hz, 1H, ArCH=C**H**$_2$); ^{13}C NMR (101 MHz, DMSO) δ 163.69, 159.47, 158.44 (d, *J* = 201.0 Hz), 149.23, 137.27, 134.70, 132.63, 132.44, 131.95, 130.37, 130.25 (d, *J* = 4.0 Hz), 129.80, 129.53 (d, *J* = 9.3 Hz), 118.09, 116.71 (d, *J* = 21.9 Hz), 112.12, 106.04.

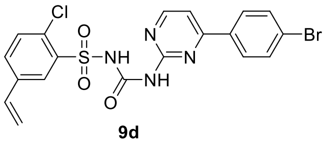

9d

***N*-((4-(4-bromophenyl)pyrimidin-2-yl)carbamoyl)-2-chloro-5-vinylbenzenesulfonamide (9d):**White solid; Yield: 78.4%; m. p.: 218-219 °C; HRMS for $C_{19}H_{14}BrClN_4O_3S$: calcd 492.9731 [M+H]$^+$, found 492.9730; ^{1}H NMR (400 MHz, DMSO) δ 13.26 (s, 1H, SO_2N**H**CONH), 10.88 (s, 1H, SO_2NHCON**H**), 8.80 (d, *J* = 4.5 Hz, 1H, pyrim-H), 8.18 (s, 1H, Ph-H), 8.11 (d, *J* = 7.9 Hz, 2H, Ph-H), 7.88 (d, *J* = 8.1 Hz, 1H, Ph-H), 7.83 (d, *J* = 4.8 Hz, 1H, pyrim-H), 7.78 (d, *J* = 7.9 Hz, 2H, Ph-H), 7.66 (d, *J* = 8.3 Hz, 1H, Ph-H), 6.90 (dd, *J* = 17.3, 11.1 Hz, 1H, ArC**H**=CH_2), 6.00 (d, *J* = 17.7 Hz, 1H, ArCH=C**H**$_2$), 5.47 (d, *J* = 10.8 Hz, 1H, ArCH=C**H**$_2$). ^{13}C NMR (101 MHz, DMSO) δ 163.62, 159.65, 157.50, 149.10, 137.26, 136.78, 134.69, 132.64, 132.44, 132.16, 130.24, 129.81, 129.68, 129.19, 126.29, 118.08, 112.24.

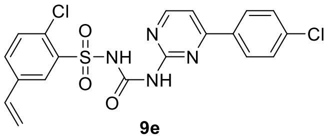

9e

2-chloro-*N*-((4-(4-chlorophenyl)pyrimidin-2-yl)carbamoyl)-5-vinylbenzenesulfonamide (9e):White solid; Yield: 77.0%; m. p.: 220-221 °C; HRMS for $C_{19}H_{14}Cl_2N_4O_3S$: calcd 449.0236 [M+H]$^+$, found 449.0238; ^{1}H NMR (400 MHz, DMSO) δ 13.28 (s, 1H, SO_2N**H**CONH), 10.89 (s, 1H, SO_2NHCON**H**), 8.82 (d, *J* = 5.1 Hz, 1H, pyrim-H), 8.22 (s, 1H, Ph-H), 8.20 (s, 2H, Ph-H), 7.90 (d, *J* = 8.3 Hz, 1H, Ph-H), 7.85 (d, *J* = 5.2 Hz, 1H, pyrim-H), 7.67 (t, *J* = 7.3 Hz, 3H, Ph-H), 6.91 (dd, *J* = 17.7, 11.1 Hz, 1H, ArC**H**=CH_2), 6.02 (d, *J* = 17.6 Hz, 1H, ArCH=C**H**$_2$), 5.48 (d, *J* = 11.0 Hz, 1H, ArCH=C**H**$_2$); ^{13}C NMR (101 MHz, DMSO) δ 163.51, 159.67, 157.50, 149.11, 137.32, 137.26, 136.85, 134.70, 134.32, 132.63, 132.44, 130.23, 129.81, 129.71, 129.52, 118.08, 112.29.

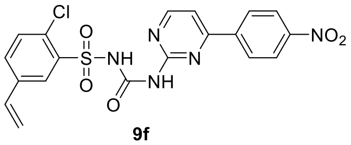

9f

2-chloro-*N*-((4-(4-nitrophenyl)pyrimidin-2-yl)carbamoyl)-5-vinylbenzenesulfonamide (9f):White solid; Yield: 80.6%; m. p.: 207-208 °C; HRMS for $C_{19}H_{14}ClN_5O_5S$: calcd 460.0477 [M+H]$^+$, found 460.0478; ^{1}H NMR (400 MHz, DMSO) δ 13.04 (s, 1H, SO_2N**H**CONH), 10.92 (s, 1H, SO_2NHCON**H**), 8.92 (d, *J* = 5.2 Hz, 1H, pyrim-H), 8.42 (s, 4H, Ph-H), 8.19 (s, 1H, Ph-H), 7.97 (d, *J* = 5.1 Hz, 1H, pyrim-H), 7.90 (d, *J* = 8.4 Hz, 1H, Ph-H), 7.69 (d, *J* = 8.2 Hz, 1H, Ph-H), 6.92 (dd, *J* = 17.5, 10.9 Hz, 1H, ArC**H**=CH_2), 6.03 (d, *J* = 17.5 Hz, 1H, ArCH=C**H**$_2$), 5.49 (d, *J* = 11.0 Hz, 1H, ArCH=C**H**$_2$); ^{13}C NMR (101 MHz, DMSO) δ

162.67, 160.07, 157.64, 149.76, 148.96, 141.48, 137.27, 134.69, 132.64, 132.46, 130.24, 129.84, 129.09, 124.60, 118.09, 113.33, 107.07.

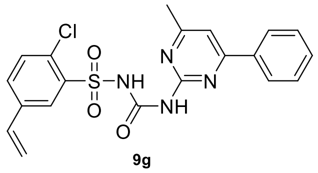

9g

chloro-*N*-((4-methyl-6-phenylpyrimidin-2-yl)carbamoyl)-5-vinylbenzenesulfonamide (9g):White solid; Yield: 69.9%; m. p.: 218-220 °C; HRMS for $C_{20}H_{17}ClN_4O_3S$: calcd 429.0783 $[M+H]^+$, found 429.0785; ^1H NMR (400 MHz, DMSO-d_6) δ 13.81 (s, 1H, SO_2N**H**CONH), 10.86 (s, 1H, SO_2NHCON**H**), 8.25 – 8.12 (m, 3H, Ph-H), 7.90 (dd, *J* = 8.38, 2.16 Hz, 1H, Ph-H), 7.78 (s, 1H, pyrim-H), 7.68 (d, *J* = 8.32 Hz, 1H, Ph-H), 7.65 – 7.54 (m, 3H, Ph-H), 6.91 (dd, *J* = 17.64, 10.98 Hz, 1H, ArC**H**=CH_2), 6.02 (d, *J* = 17.63 Hz, 1H, ArCH=C**H**$_2$), 5.48 (d, *J* = 10.97 Hz, 1H, ArCH=C**H**$_2$), 2.53 (s, 3H, pyrim-CH_3); ^{13}C NMR (101 MHz, DMSO) δ 170.12, 163.77, 157.31, 149.23, 137.28, 136.75, 135.44, 134.69, 132.65, 132.48, 132.27, 130.25, 129.73, 129.62, 127.60, 118.10, 111.78, 24.17.

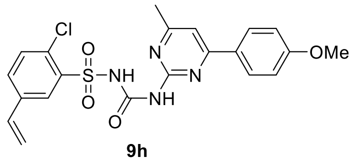

9h

2-chloro-*N*-((4-(4-methoxyphenyl)-6-methylpyrimidin-2-yl)carbamoyl)-5-vinylbenzenesulfonamide (9h):White solid; Yield: 60.5%; m. p.: 160-162 °C; HRMS for $C_{21}H_{19}ClN_4O_4S$: calcd 459.0888 $[M+H]^+$, found 459.0889; ^1H NMR (400 MHz, DMSO-d_6) δ 13.97 (s, 1H, SO_2N**H**CONH), 10.80 (s, 1H, SO_2NHCON**H**), 8.18 (d, *J* = 7.80 Hz, 2H, Ph-H), 8.15 (s, 1H, pyrim-H), 7.89 (d, *J* = 8.30 Hz, 1H, Ph-H), 7.74 – 7.64 (m, 2H, Ph-H), 7.11 (d, *J* = 8.44 Hz, 2H, Ph-H), 6.91 (dd, *J* = 17.64, 10.95 Hz, 1H, ArC**H**=CH_2), 6.02 (d, *J* = 17.61 Hz, 1H, ArCH=C**H**$_2$), 5.48 (d, *J* = 10.93 Hz, 1H, ArCH=C**H**$_2$), 3.86 (s, 3H, Ph-OCH_3), 2.51 (s, 3H, pyrim-CH_3); ^{13}C NMR (101 MHz, DMSO) δ 168.86, 162.90, 162.22, 156.65, 148.85, 136.72, 136.31, 134.17, 132.09, 131.89, 129.70, 129.23, 128.85, 126.99, 117.51, 114.43, 110.18, 55.45, 23.56.

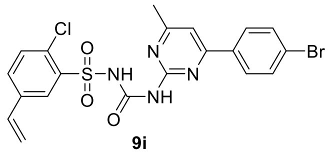

9i

***N*-((4-(4-bromophenyl)-6-methylpyrimidin-2-yl)carbamoyl)-2-chloro-5-vinylbenzenesulfonamide (9i):**White solid; Yield: 77.3%; m. p.: 212-214 °C; HRMS for $C_{20}H_{16}BrClN_4O_3S$: calcd 506.9888 $[M+H]^+$, found 506.9891; ^1H NMR (400 MHz, DMSO) δ 13.62 (s, 1H, SO_2N**H**CONH), 10.83 (s, 1H, SO_2NHCON**H**), 8.18 (s, 1H, pyrim-H), 8.10 (d, *J* = 7.6 Hz, 2H, Ph-H), 7.89 (d, *J* = 8.3 Hz, 1H, Ph-H), 7.80 (s, 1H, Ph-H), 7.79 (s, 2H, Ph-H), 7.68 (d, *J* = 8.0 Hz, 1H, Ph-H), 6.91 (dd, *J* = 17.5, 10.8 Hz, 1H, ArC**H**=CH_2), 6.02 (d, *J* = 17.6 Hz, 1H, ArCH=C**H**$_2$), 5.48 (d, *J* = 11.0 Hz, 1H, ArCH=C**H**$_2$), 2.53 (s, 3H, pyrim-CH_3); ^{13}C NMR (101 MHz, DMSO) δ 169.51, 162.50, 156.81, 148.62, 136.77, 136.19, 134.28, 134.18, 132.16, 132.10, 131.98, 129.74, 129.24, 129.06, 125.54, 117.61, 111.26, 23.66.

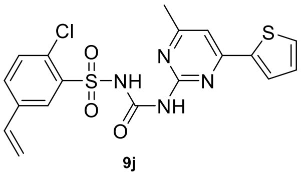

9j

2-chloro-*N*-((4-methyl-6-(thiophen-2-yl)pyrimidin-2-yl)carbamoyl)-5-vinylbenzenesulfonamide (9j):White solid; Yield: 64.2%; m. p.: 215-217 °C; HRMS for $C_{18}H_{15}ClN_4O_3S_2$: calcd 435.0347 $[M+H]^+$, found 435.0349; ^1H NMR (400 MHz, DMSO) δ 13.34 (s, 1H, SO_2N**H**CONH), 10.81 (s, 1H, SO_2NHCON**H**), 8.18 (s, 1H, pyrim-H), 8.10 (s, 1H, thienyl-H), 7.95 (d, *J* = 4.4 Hz, 1H, thienyl-H), 7.89 (d, *J* = 8.2 Hz, 1H, Ph-H), 7.72 – 7.63 (m, 2H, Ph-H), 7.30 (s, 1H, thienyl-H), 6.91 (dd, *J* = 17.6, 11.1 Hz, 1H, ArCH=C**H**$_2$), 6.01 (d, *J* = 17.6 Hz, 1H, ArCH=C**H**$_2$), 5.48 (d, *J* = 10.9 Hz, 1H, ArCH=C**H**$_2$), 2.48 (s, 3H, pyrim-CH_3); ^{13}C NMR (101 MHz, DMSO) δ 169.57, 158.93, 157.06, 149.15, 140.80, 137.23, 136.79, 134.69, 132.61, 132.49, 132.45, 130.29, 130.12, 129.79, 129.64, 118.06, 109.95, 24.03.

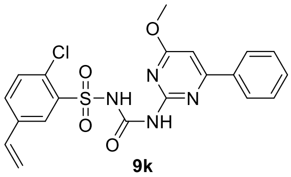

9k

2-chloro-*N*-((4-methoxy-6-phenylpyrimidin-2-yl)carbamoyl)-5-vinylbenzenesulfonamide (9k):White solid; Yield: 60.8%; m. p.: 193-195 °C; HRMS for $C_{20}H_{17}ClN_4O_4S$: calcd 445.0732 $[M+H]^+$, found 445.0733;^1H NMR (400 MHz, DMSO) δ 13.87 (s, 1H, SO_2N**H**CONH), 10.84 (s, 1H, SO_2NHCON**H**), 8.19 (s, 1H, Ph-H), 8.15 (d, *J* = 7.3 Hz, 2H, Ph-H), 7.90 (d, *J* = 8.1 Hz, 1H, Ph-H), 7.68 (d, *J* = 8.2 Hz, 1H), 7.62 – 7.52 (m, 3H, Ph-H), 7.27 (s, 1H, pyrim-H), 6.91 (dd, *J* = 17.7, 11.0 Hz, 1H, ArC**H**=CH_2), 6.02 (d, *J* = 17.7 Hz, 1H, ArCH=C**H**$_2$), 5.49 (d, *J* = 10.9 Hz, 1H, ArCH=C**H**$_2$), 4.00 (s, 3H, pyrim-OCH_3). ^{13}C NMR (101 MHz, DMSO) δ 171.72, 163.90, 157.30, 149.20, 137.25, 136.68, 135.22, 134.65, 132.59, 132.43, 131.98, 130.31, 129.78, 129.47, 127.42, 118.03, 98.42, 54.97.

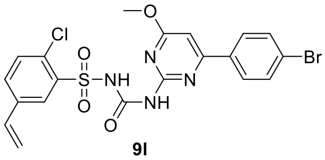

9l

***N*-((4-(4-bromophenyl)-6-methoxypyrimidin-2-yl)carbamoyl)-2-chloro-5-vinylbenzenesulfonamide (9l):**White solid; Yield: 71.1%; m. p.: 201-202 °C; HRMS for $C_{20}H_{16}BrClN_4O_4S$: calcd 522.9837 $[M+H]^+$, found 522.9841; ^1H NMR (400 MHz, DMSO) δ 13.58 (s, 1H, SO_2N**H**CONH), 10.83 (s, 1H, SO_2NHCON**H**), 8.20 (s, 1H, Ph-H), 8.07 (d, *J* = 7.2 Hz, 2H, Ph-H), 7.89 (d, *J* = 8.2 Hz, 1H, Ph-H), 7.75 (d, *J* = 8.0 Hz, 2H, Ph-H), 7.68 (d, *J* = 8.2 Hz, 1H, Ph-H), 7.27 (s, 1H, pyrim-H), 6.91 (dd, *J* = 17.2, 11.2 Hz, 1H, ArC**H**=CH_2), 6.02 (d, *J* = 17.8 Hz, 1H, ArCH=C**H**$_2$), 5.49 (d, *J* = 10.7 Hz, 1H, ArCH=C**H**$_2$), 4.01 (s, 3H, pyrim-OCH_3); ^{13}C NMR (101 MHz, DMSO) δ 171.68, 163.16, 159.40, 157.29, 149.00, 137.29, 136.59, 134.67, 132.65, 132.51, 132.46, 130.32, 129.78, 129.44, 125.70, 118.12, 98.61, 55.10.

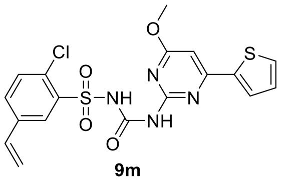

9m

2-chloro-*N*-((4-methoxy-6-(thiophen-2-yl)pyrimidin-2-yl)carbamoyl)-5-vinylbenzenesulfonamide (9m):White solid; Yield: 66.9%; m. p.:188-189 °C; HRMS for $C_{18}H_{15}ClN_4O_4S_2$: calcd 451.0296 [M+H]$^+$, found 451.0298; ^{1}H NMR (400 MHz, DMSO) δ 13.20 (s, 1H, SO$_2$N**H**CONH), 10.80 (s, 1H, SO$_2$NHCON**H**), 8.22 (d, *J* = 1.3 Hz, 1H, Ph-H), 8.10 (d, *J* = 3.2 Hz, 1H, Ph-H), 7.92 (d, *J* = 4.8 Hz, 1H, thienyl-H), 7.89 (d, *J* = 8.4 Hz, 1H, thienyl-H), 7.68 (d, *J* = 8.3 Hz, 1H, thienyl-H), 7.28 (t, *J* = 4.00 Hz, 1H, Ph-H), 7.17 (s, 1H, pyrim-H), 6.91 (dd, *J* = 17.6, 11.0 Hz, 1H, ArC**H**=CH$_2$), 6.02 (d, *J* = 17.6 Hz, 1H, ArCH=C**H**$_2$), 5.49 (d, *J* = 11.0 Hz, 1H, ArCH=C**H**$_2$), 3.99 (s, 3H, pyrim-OCH$_3$); ^{13}C NMR (101 MHz, DMSO) δ 171.43, 159.07, 157.12, 149.01, 140.57, 137.25, 136.68, 134.67, 132.60, 131.74, 130.38, 129.56, 129.35, 128.65, 125.76, 118.06, 96.26, 54.98.

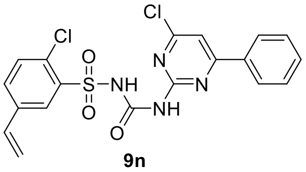

9n

2-chloro-*N*-((4-chloro-6-phenylpyrimidin-2-yl)carbamoyl)-5-vinylbenzenesulfonamide (9n):White solid; Yield: 63.8%; m. p.: 197-198 °C; HRMS for $C_{19}H_{14}Cl_2N_4O_3S$: calcd 449.0236 [M+H]$^+$, found 449.0240; ^{1}H NMR (400 MHz, DMSO) δ 12.99 (s, 1H, SO$_2$N**H**CONH), 11.05 (s, 1H, SO$_2$NHCON**H**), 8.23 (s, 1H, Ph-H), 8.20 (s, 2H, Ph-H), 8.03 (s, 1H, pyrim-H), 7.91 (d, *J* = 8.2 Hz, 1H, Ph-H), 7.65 (m, 4H, Ph-H), 6.92 (dd, *J* = 17.4, 11.0 Hz, 1H, ArC**H**=CH$_2$), 6.02 (d, *J* = 17.7 Hz, 1H, ArCH=C**H**$_2$), 5.49 (d, *J* = 10.9 Hz, 1H, ArCH=C**H**$_2$); ^{13}C NMR (101 MHz, DMSO) δ 165.62, 162.63, 157.38, 148.54, 137.33, 136.52, 134.64, 134.41, 132.99, 132.70, 132.60, 130.29, 129.79, 129.72, 127.98, 118.18, 112.13.

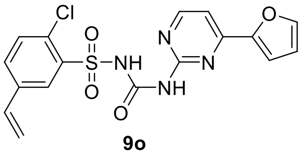

9o

chloro-*N*-((4-(furan-2-yl)pyrimidin-2-yl)carbamoyl)-5-vinylbenzenesulfonamide (9o):White solid; Yield: 55.8%; m. p.: 219-220 °C; HRMS for $C_{17}H_{13}ClN_4O_4S$: calcd 405.0419 [M+H]$^+$, found 405.0422; ^{1}H NMR (400 MHz, DMSO) δ 13.53 (s, 1H, SO$_2$N**H**CONH), 10.95 (s, 1H, SO$_2$NHCON**H**), 8.74 (d, *J* = 4.7 Hz, 1H, pyrim-H), 8.19 (s, 1H, Ph-H), 8.08 (s, 1H, pyrim-H), 7.90 (d, *J* = 8.1 Hz, 1H, Ph-H), 7.68 (d, *J* = 8.2 Hz, 1H, Ph-H), 7.51 (d, *J* = 4.9 Hz, 1H, furanyl-H), 7.48 (s, 1H, furanyl-H), 6.92 (dd, *J* = 17.5, 11.1 Hz, 1H, ArC**H**=CH$_2$), 6.86 (s, 1H, furanyl-H), 6.02 (d, *J* = 17.5 Hz, 1H, ArCH=C**H**$_2$), 5.49 (d, *J* = 10.8 Hz, 1H, ArCH=C**H**$_2$); ^{13}C NMR (101 MHz, DMSO) δ 159.69, 156.79, 154.23, 149.71, 147.41, 141.17, 141.13, 136.76, 134.19, 132.14, 131.96, 129.75, 129.27, 117.60, 114.23, 113.27, 109.34.

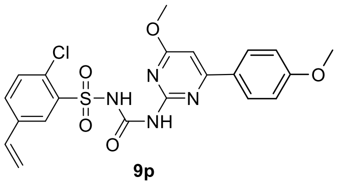

9p

chloro-N-((4-methoxy-6-(4-methoxyphenyl)pyrimidin-2-yl)carbamoyl)-5-vinylbenzenesulfonamide (9p): White solid; Yield: 81.6%; m. p.: 199-200 °C; HRMS for $C_{18}H_{15}ClN_4O_4S_2$: calcd 475.0837 $[M+H]^+$, found 475.0837; ^{1}H NMR (400 MHz, DMSO) δ 14.01 (s, 1H, SO_2N**H**CONH), 10.79 (s, 1H, SO_2NHCON**H**), 8.19 (s, 1H, Ph-H), 8.13 (d, *J* = 8.0 Hz, 2H, Ph-H), 7.90 (d, *J* = 8.1 Hz, 1H, Ph-H), 7.68 (d, *J* = 8.9 Hz, 1H, Ph-H), 7.19 (s, 1H, pyrim-H), 7.08 (d, *J* = 7.8 Hz, 2H, Ph-H), 6.91 (dd, *J* = 17.5, 11.2 Hz, 1H, ArC**H**=CH_2), 6.02 (d, *J* = 17.6 Hz, 1H, ArCH=C**H**$_2$), 5.48 (d, *J* = 11.1 Hz, 1H, ArCH=C**H**$_2$), 3.98 (s, 3H, pyrim-OCH_3), 3.85 (s, 3H, Ph-OCH_3); ^{13}C NMR (101 MHz, DMSO) δ 171.66, 163.53, 162.51, 157.19, 149.24, 137.27, 136.72, 134.69, 132.64, 132.46, 130.30, 129.76, 129.20, 127.41, 118.10, 114.85, 97.12, 55.96, 54.93.

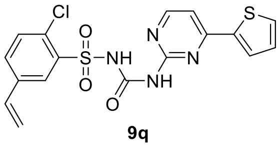

9q

2-chloro-*N*-((4-(thiophen-2-yl)pyrimidin-2-yl)carbamoyl)-5-vinylbenzenesulfonamide (9q):White solid; Yield: 70.3%; m. p.: 218-219 °C; HRMS for $C_{17}H_{13}ClN_4O_3S_2$: calcd 421.0190 $[M+H]^+$, found 421.0189; ^{1}H NMR (400 MHz, DMSO) δ 13.06 (s, 1H, SO_2N**H**CONH), 10.80 (s, 1H, SO_2NHCON**H**), 8.69 (d, *J* = 5.3 Hz, 1H, pyrim-H), 8.18 (d, *J* = 1.9 Hz, 1H, Ph-H), 8.15 (d, *J* = 3.6 Hz, 1H, thienyl-H), 7.97 (d, *J* = 4.9 Hz, 1H, thienyl-H), 7.88 (d, *J* = 7.2 Hz, 1H, Ph-H), 7.72 (d, *J* = 5.4 Hz, 1H, pyrim-H), 7.67 (d, *J* = 8.3 Hz, 1H, Ph-H), 7.31(t, *J* = 4.40 Hz, 1H, thienyl-H), 6.91 (dd, *J* = 17.7, 11.0 Hz, 1H, ArC**H**=CH_2), 6.01 (d, *J* = 17.7 Hz, 1H, ArCH=C**H**$_2$), 5.47 (d, *J* = 10.0 Hz, 1H, ArCH=C**H**$_2$); ^{13}C NMR (101 MHz, DMSO) δ 159.56, 159.18, 157.30, 149.24, 140.78, 137.17, 134.75, 132.97, 132.55, 132.28, 130.78, 130.24, 129.85, 129.72, 128.88, 117.98, 110.58.

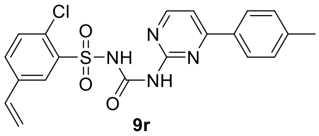

9r

2-chloro-*N*-((4-(p-tolyl)pyrimidin-2-yl)carbamoyl)-5-vinylbenzenesulfonamide (9r):White solid; Yield: 79.0%; m. p.: 216-217 °C; HRMS for $C_{20}H_{17}ClN_4O_3S$: calcd 429.0783 $[M+H]^+$, found 429.0781; ^{1}H NMR (400 MHz, DMSO) δ 13.55 (s, 1H, SO_2N**H**CONH), 10.89 (s, 1H, SO_2NHCON**H**), 8.76 (d, *J* = 4.6 Hz, 1H, pyrim-H), 8.20 (s, 1H, Ph-H), 8.11 (d, *J* = 7.7 Hz, 2H, Ph-H), 7.89 (d, *J* = 8.0 Hz, 1H, Ph-H), 7.80 (d, *J* = 5.1 Hz, 1H, pyrim-H), 7.67 (d, *J* = 8.2 Hz, 1H, Ph-H), 7.40 (d, *J* = 7.6 Hz, 2H, Ph-H), 6.91 (dd, *J* = 17.5, 11.1 Hz, 1H, ArC**H**=CH_2), 6.02 (d, *J* = 17.7 Hz, 1H, ArCH=C**H**$_2$), 5.48 (d, *J* = 10.9 Hz, 1H, ArCH=C**H**$_2$), 2.41 (s, 3H, Ph-CH_3); ^{13}C NMR (101 MHz, DMSO) δ 163.78, 159.13, 156.93, 148.74, 142.31, 136.75, 136.31, 134.21, 132.13, 131.98, 131.91, 129.78, 129.28, 129.25, 127.17, 117.58, 111.37, 20.98.

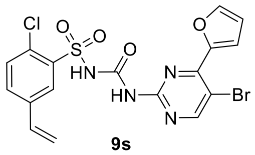

9s

***N*-((5-bromo-4-(furan-2-yl)pyrimidin-2-yl)carbamoyl)-2-chloro-5-vinylbenzenesulfonamide (9s):** White solid; Yield: 71.4%; m. p.: 226-227 °C; HRMS for $C_{17}H_{12}BrClN_4O_4S$: calcd 482.9524 [M+H]$^+$, found 482.9522; ^{1}H NMR (400 MHz, DMSO-*d*$_6$) δ 13.04 (s, 1H, SO$_2$N**H**CONH), 11.00 (s, 1H, SO$_2$NHCON**H**), 8.93 (s, 1H, pyrim-H), 8.18 (d, *J* = 2.14 Hz, 1H, furanyl-H), 8.10 (s, 1H, Ph-H), 7.89 (d, *J* = 8.35, 1H, Ph-H), 7.80 (d, *J* = 3.67 Hz, 1H, furanyl-H), 7.68 (d, *J* = 8.32 Hz, 1H, Ph-H), 6.95 – 6.85 (m, 2H, ArC**H**=CH$_2$ & furanyl-H), 6.01 (d, *J* = 17.66 Hz, 1H, ArCH=C**H**$_2$), 5.48 (d, *J* = 11.00 Hz, 1H, ArCH=C**H**$_2$); ^{13}C NMR (101 MHz, DMSO) δ 162.73, 155.13, 150.77, 148.39, 147.94, 147.37, 136.71, 136.23, 134.17, 132.13, 131.95, 129.72, 129.40, 118.38, 117.57, 113.20, 106.18.

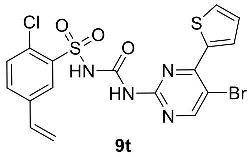

9t

***N*-((5-bromo-4-(thiophen-2-yl)pyrimidin-2-yl)carbamoyl)-2-chloro-5-vinylbenzenesulfonamide (9t):** White solid; Yield: 61.3%; m. p.: 228-230 °C; HRMS for $C_{17}H_{12}BrClN_4O_3S_2$: calcd 498.9295 [M+H]$^+$, found 498.9298; ^{1}H NMR (400 MHz, DMSO) δ 12.34 (s, 1H, SO$_2$N**H**CONH), 10.85 (s, 1H, SO$_2$NHCON**H**), 8.95 (s, 1H, pyrim-H), 8.41 (d, *J* = 3.6 Hz, 1H, thienyl-H), 8.18 (s, 1H, Ph-H), 8.08 (d, *J* = 5.0 Hz, 1H, thienyl-H), 7.90 (d, *J* = 8.3 Hz, 1H, Ph-H), 7.69 (d, *J* = 8.2 Hz, 1H, Ph-H), 7.35 (t, *J* = 4.1 Hz, 1H, thienyl-H), 6.91 (dd, *J* = 17.7, 11.1 Hz, 1H, ArC**H**=CH$_2$), 6.02 (d, *J* = 17.7 Hz, 1H, ArCH=C**H**$_2$), 5.48 (d, *J* = 10.9 Hz, 1H, ArCH=C**H**$_2$); ^{13}C NMR (101 MHz, DMSO) δ 162.93, 156.03, 155.54, 148.58, 139.52, 137.26, 136.70, 134.67, 133.94, 133.11, 132.64, 132.50, 130.31, 129.86, 129.34, 118.12, 108.09.

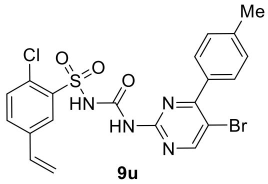

9u

((5-bromo-4-(4-methoxyphenyl)pyrimidin-2-yl)carbamoyl)-2-chloro-5-vinylbenzenesulfonamide (9u): White solid; Yield: 66.8%; m. p.: 214-215 °C; HRMS for $C_{20}H_{16}BrClN_4O_4S$: calcd 506.9888 [M+H]$^+$, found 506.9892; ^{1}H NMR (400 MHz, DMSO) δ 12.69 (s, 1H, SO$_2$N**H**CONH), 10.95 (s, 1H, SO$_2$NHCON**H**), 8.99 (s, 1H, pyrim-H), 8.16 (s, 1H, Ph-H), 7.89 (d, *J* = 7.9 Hz, 1H, Ph-H), 7.72 (d, *J* = 7.8 Hz, 2H, Ph-H), 7.67 (d, *J* = 8.3 Hz, 1H, Ph-H), 7.38 (d, *J* = 7.8 Hz, 2H, Ph-H), 6.90 (dd, *J* = 17.6, 11.0 Hz, 1H, ArC**H**=CH$_2$), 6.00 (d, *J* = 17.6 Hz, 1H, ArCH=C**H**$_2$), 5.47 (d, *J* = 11.0 Hz, 1H, ArCH=C**H**$_2$), 2.41 (s, 3H, Ph-CH$_3$); ^{13}C NMR (101 MHz, DMSO) δ 164.21, 161.92, 156.12, 148.69, 141.20, 137.24, 136.63, 134.65, 133.49, 132.63, 132.47, 130.20, 129.85, 129.49, 129.38, 118.09, 111.20, 21.48.

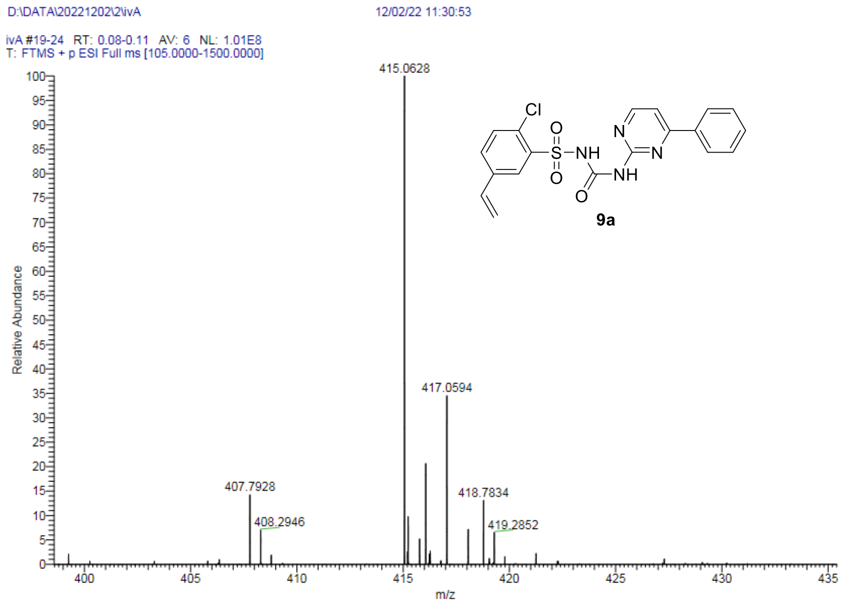

HRMS spectra for compound 9a.

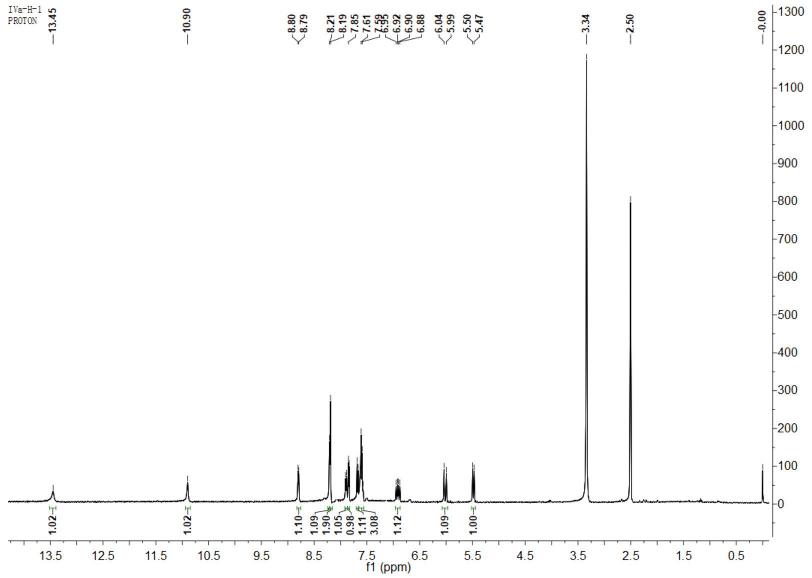

¹H NMR spectra for compound 9a.

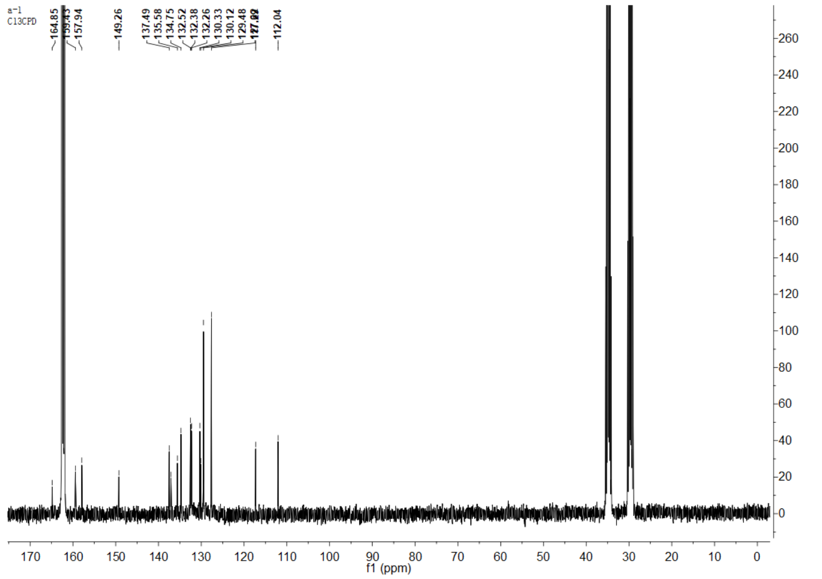

^{13}C NMR spectra for compound 9a.

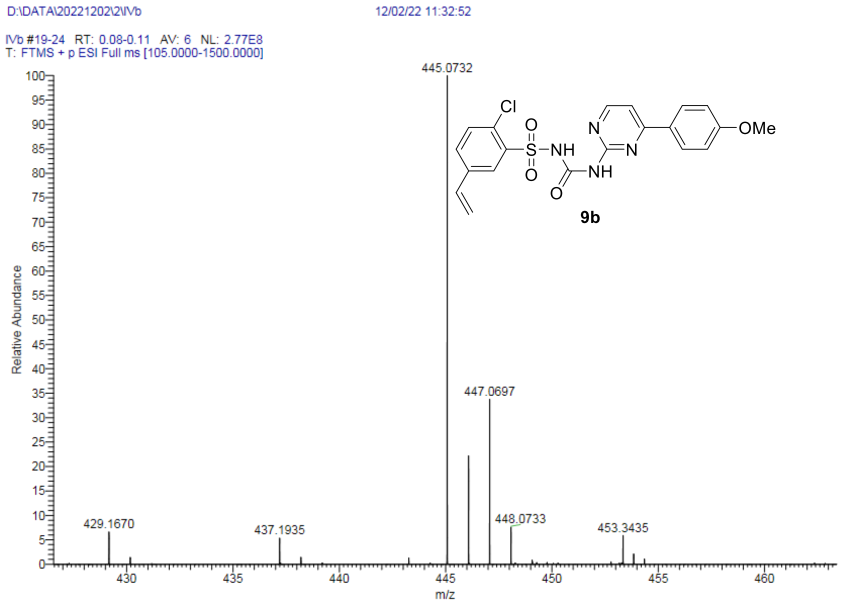

HRMS spectra for compound 9b.

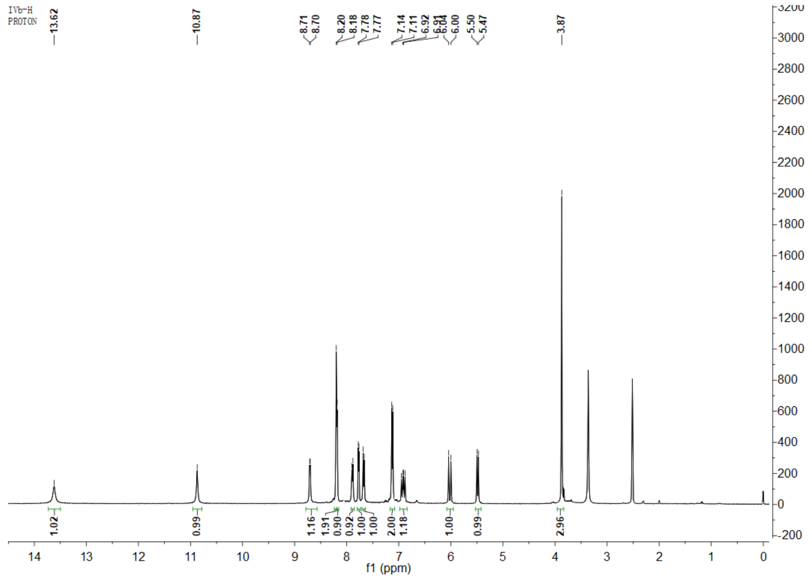

^{1}H NMR spectra for compound 9b.

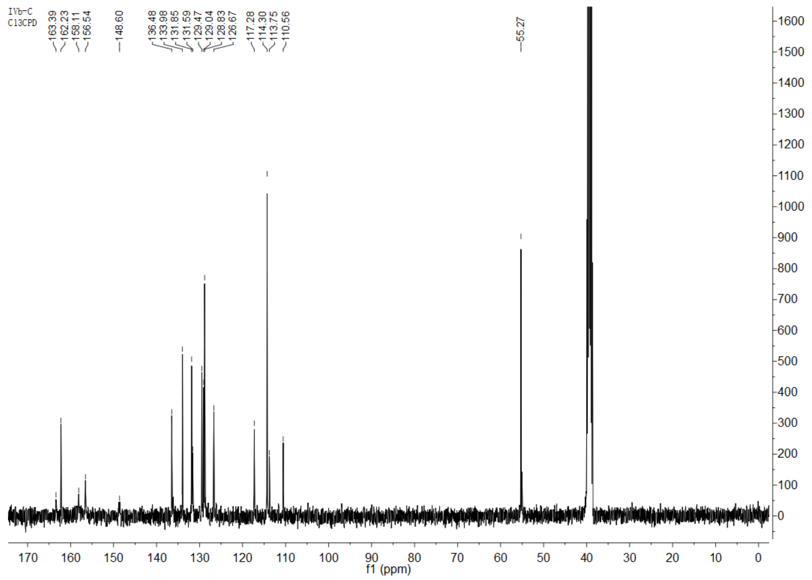

^{13}C NMR spectra for compound 9b.

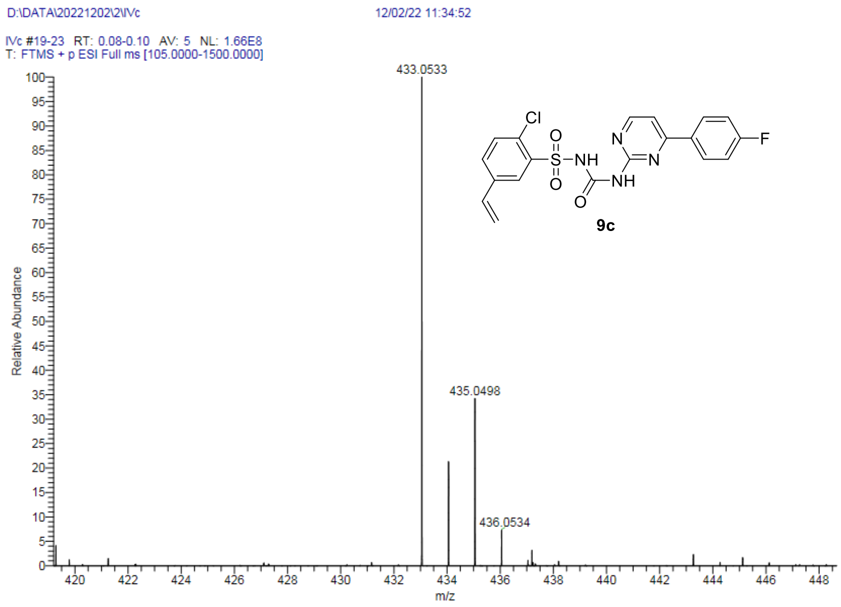

HRMS spectra for compound 9c.

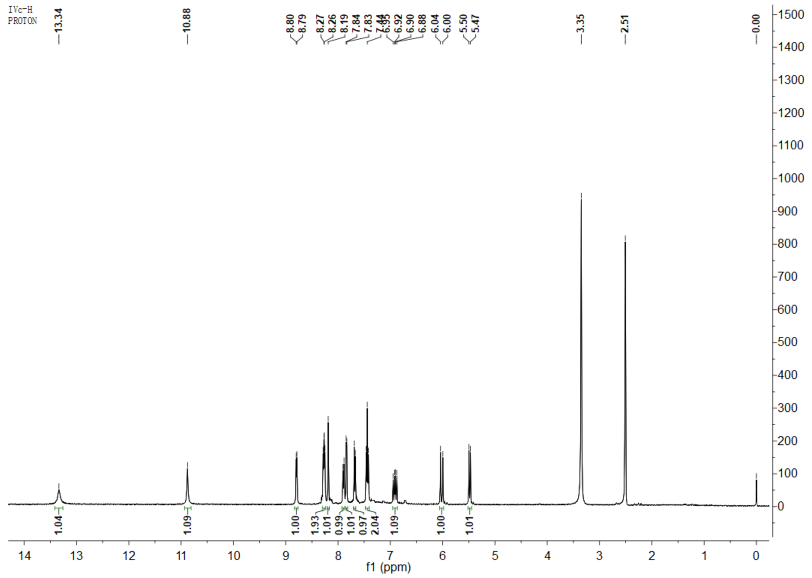

^{1}H NMR spectra for compound 9c.

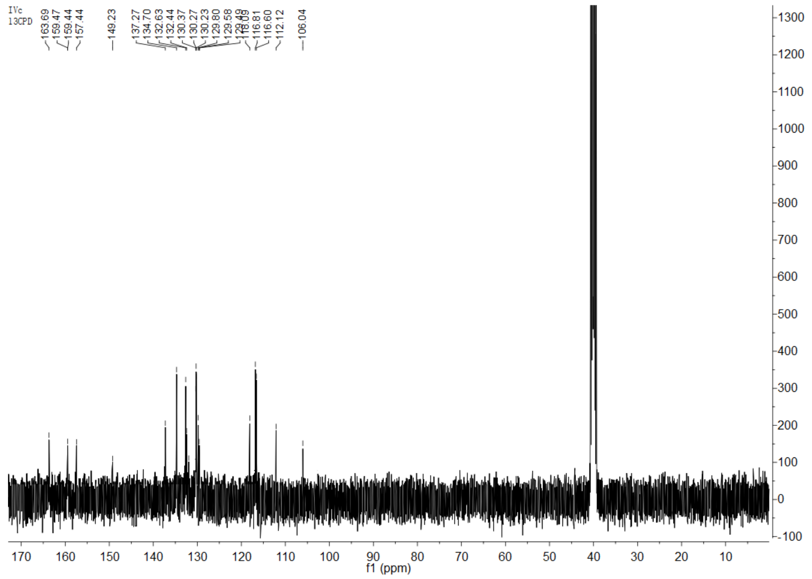

^{13}C NMR spectra for compound 9c.

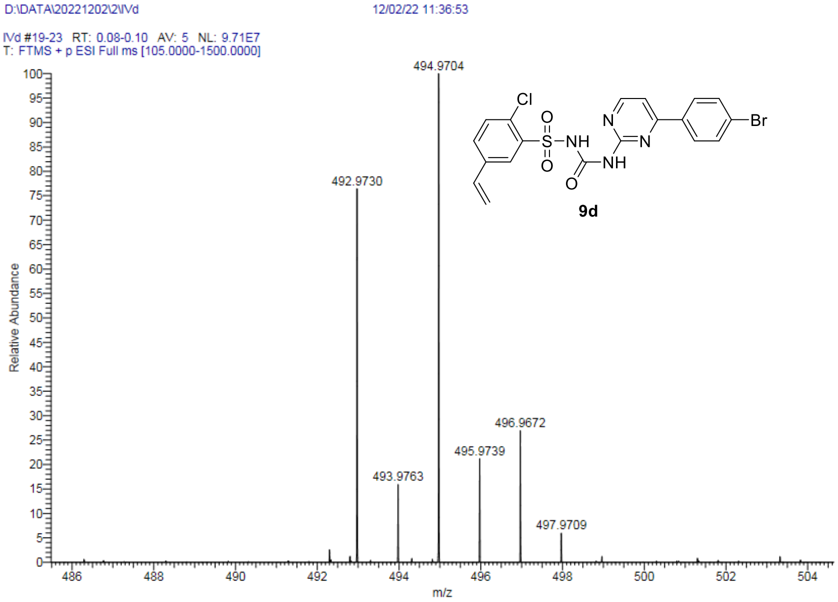

HRMS spectra for compound 9d.

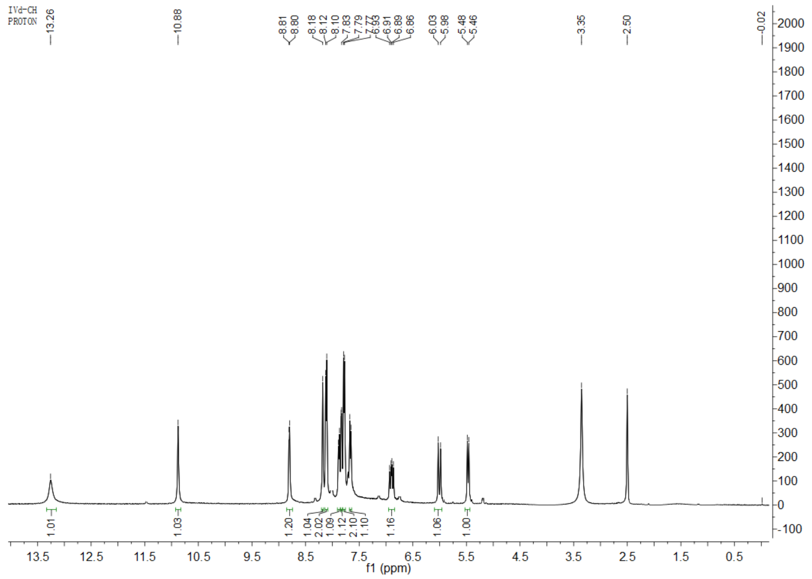

¹H NMR spectra for compound 9d.

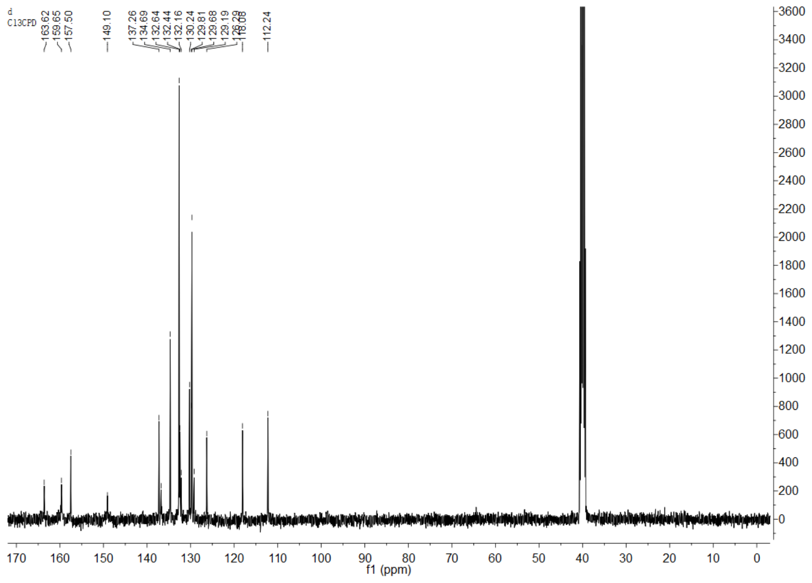

¹³C NMR spectra for compound 9d.

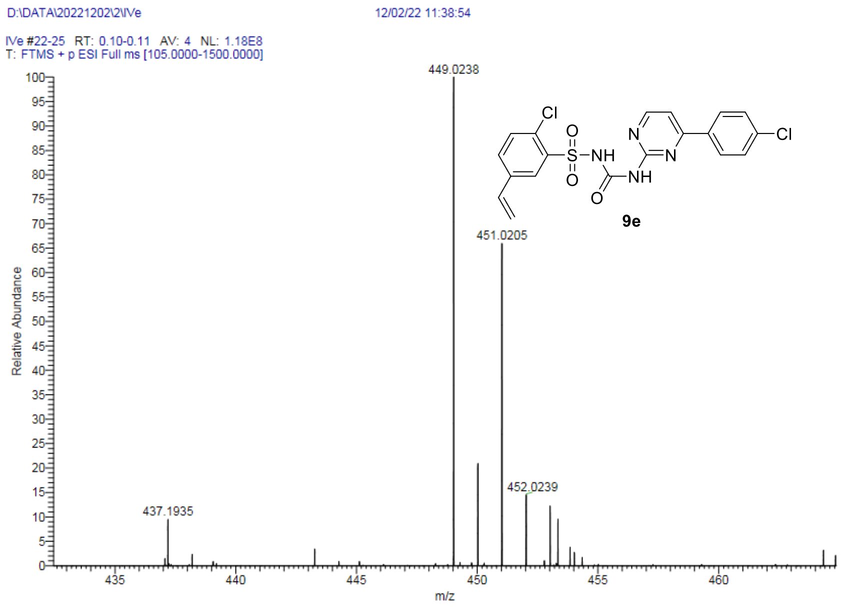

HRMS spectra for compound 9e.

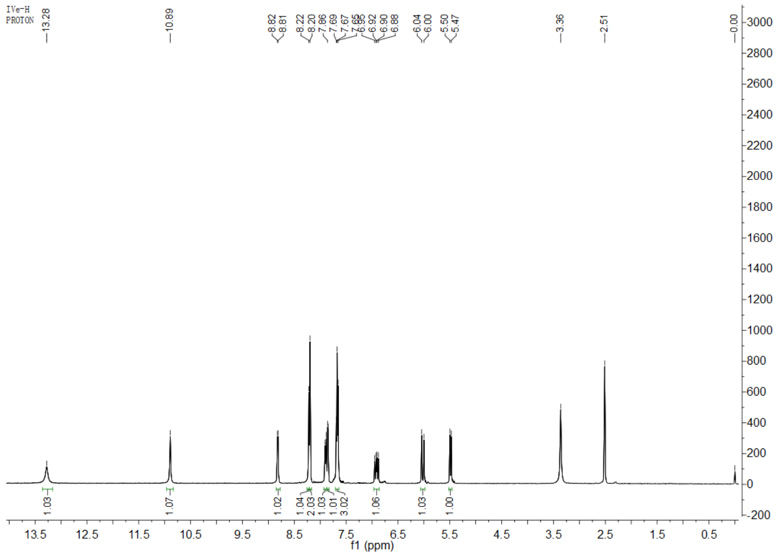

^{1}H NMR spectra for compound 9e.

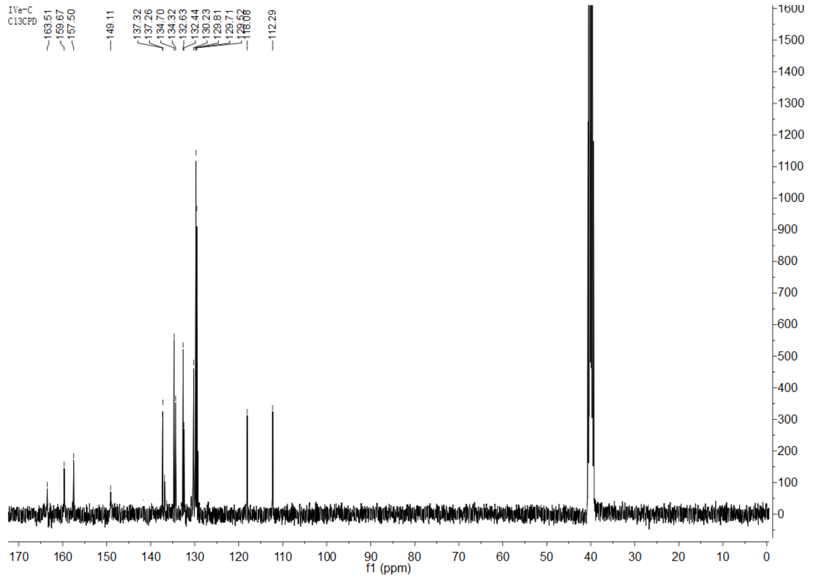

^{13}C NMR spectra for compound 9e.

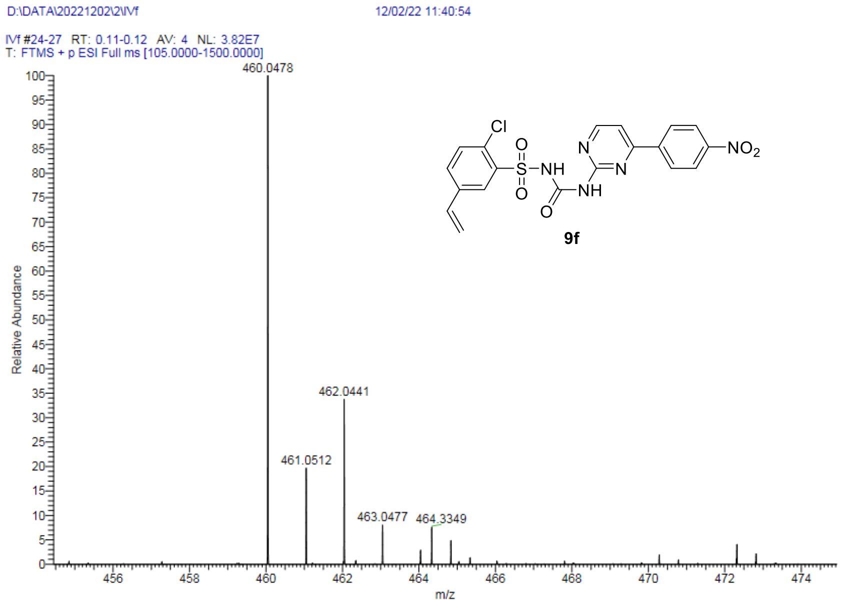

HRMS spectra for compound 9f.

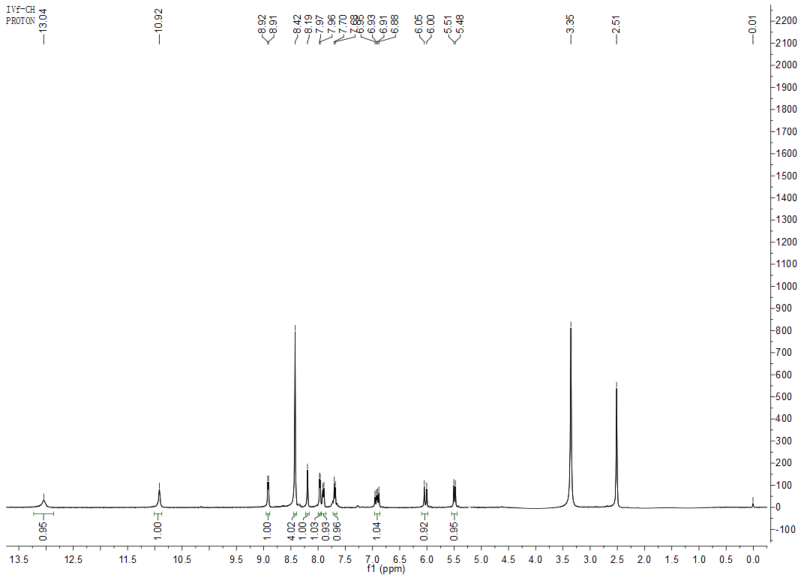

¹H NMR spectra for compound 9f.

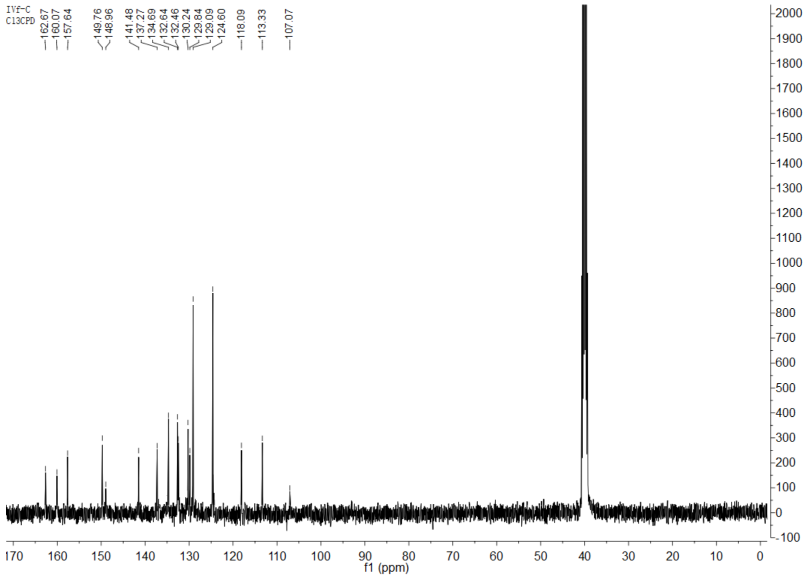

¹³C NMR spectra for compound 9f.

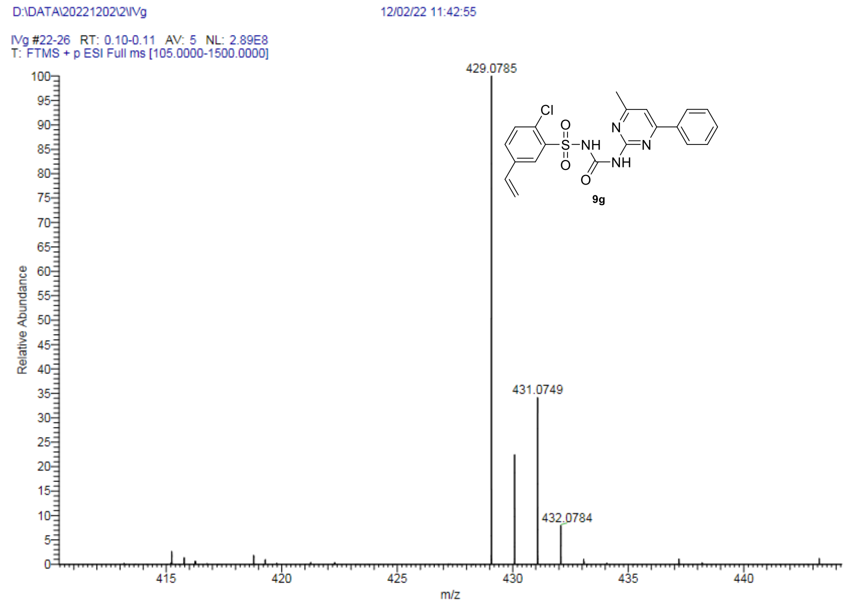

HRMS spectra for compound 9g.

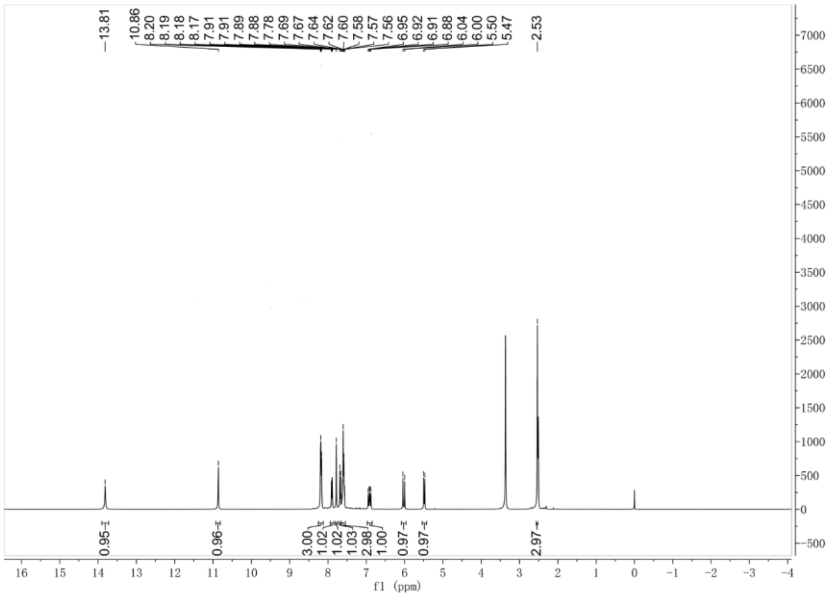

^{1}H NMR spectra for compound 9g.

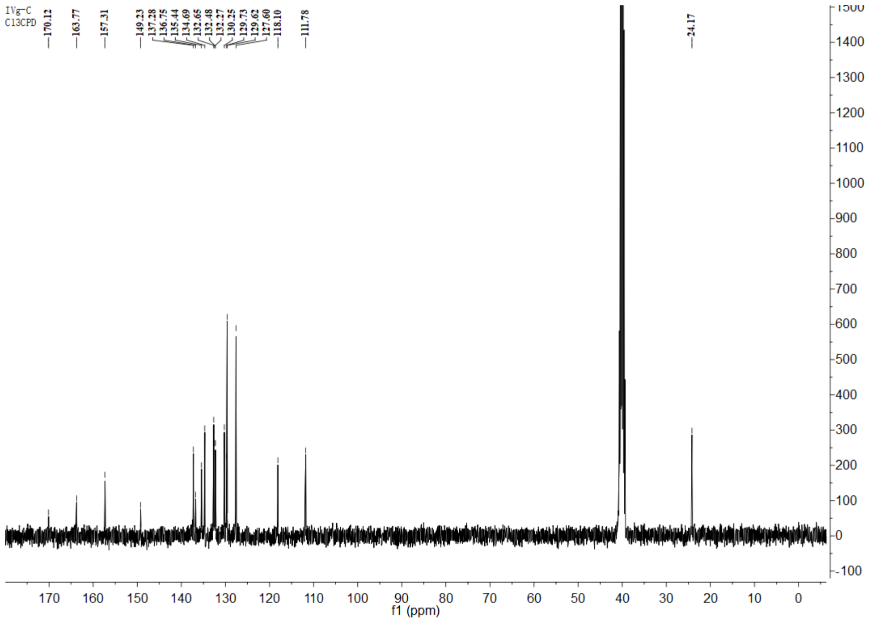

^{13}C NMR spectra for compound 9g.

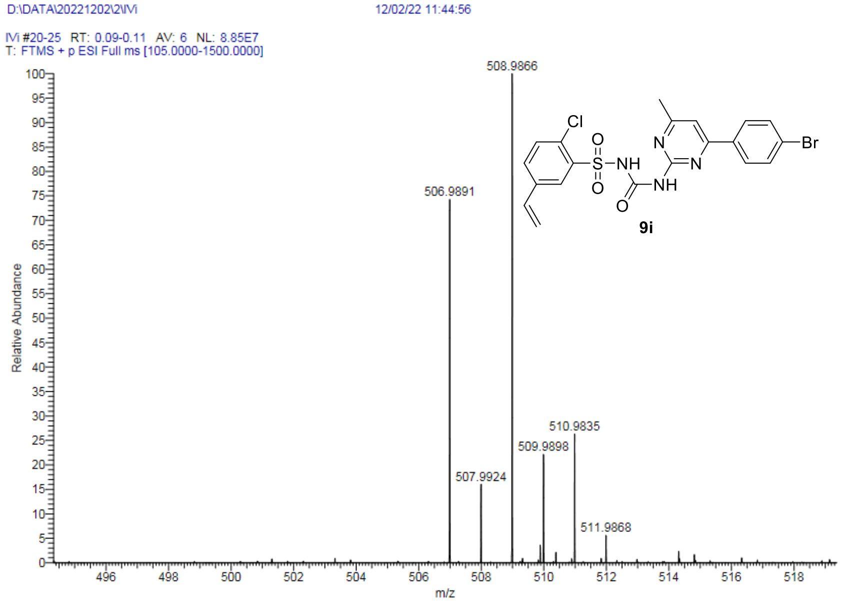

HRMS spectra for compound 9i.

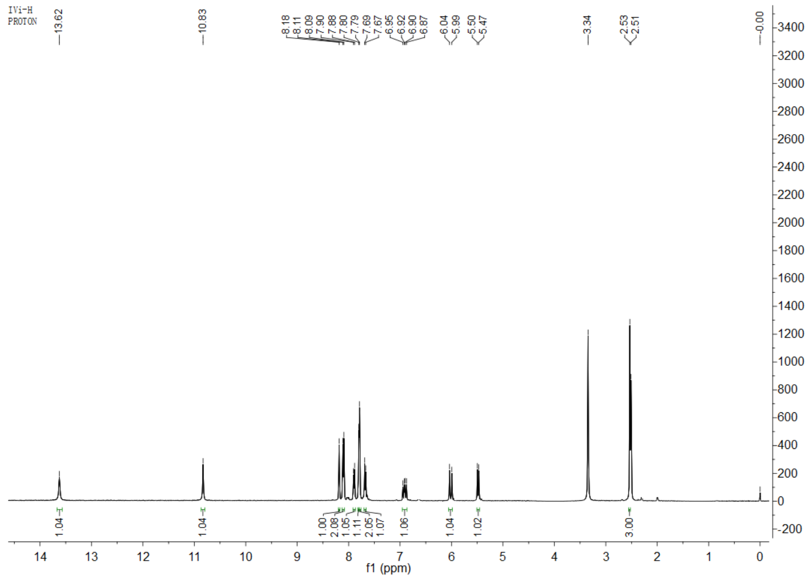

¹H NMR spectra for compound 9i.

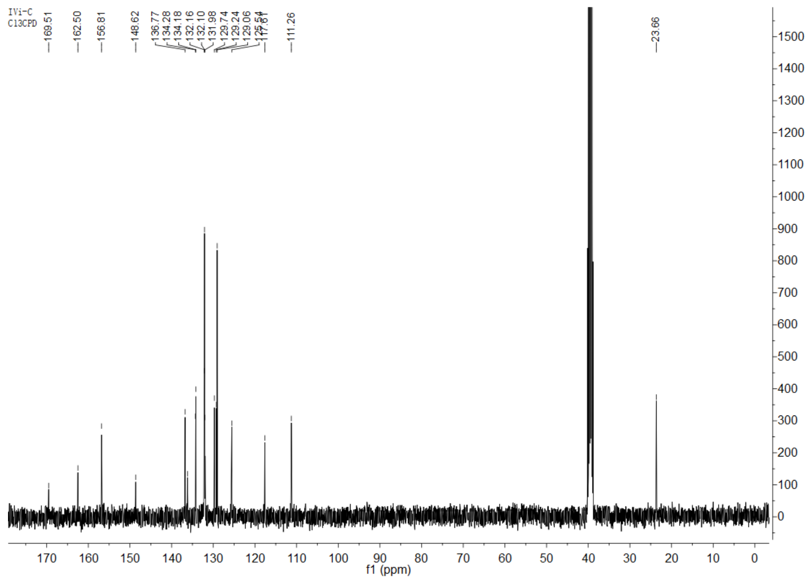

¹³C NMR spectra for compound 9i.

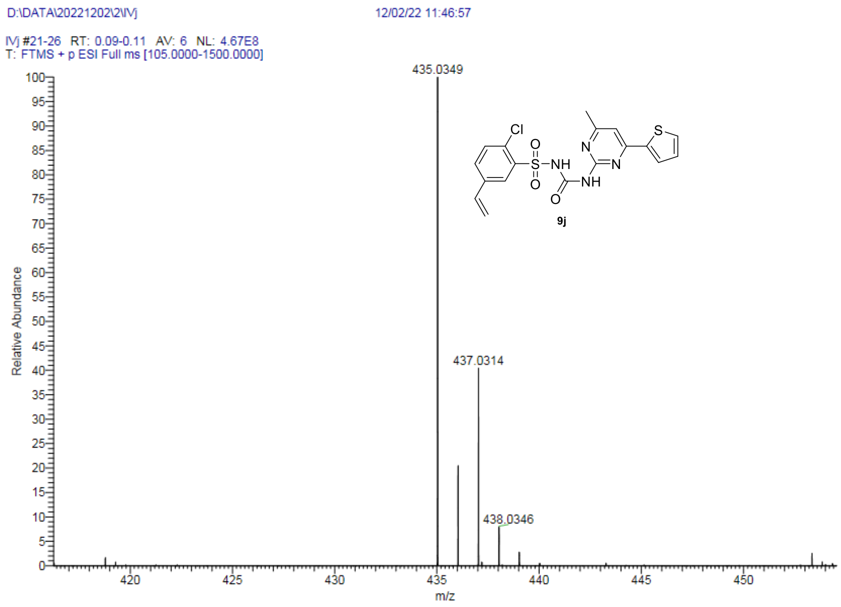

HRMS spectra for compound 9j.

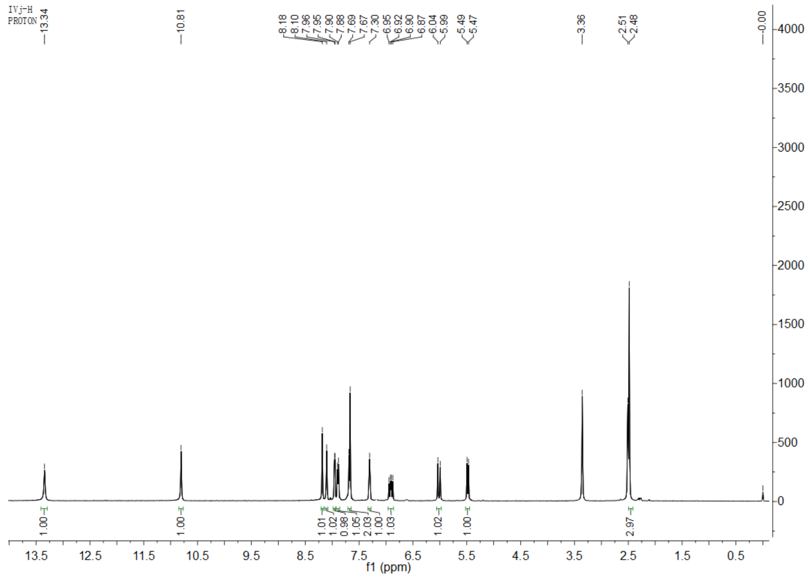

¹H NMR spectra for compound 9j.

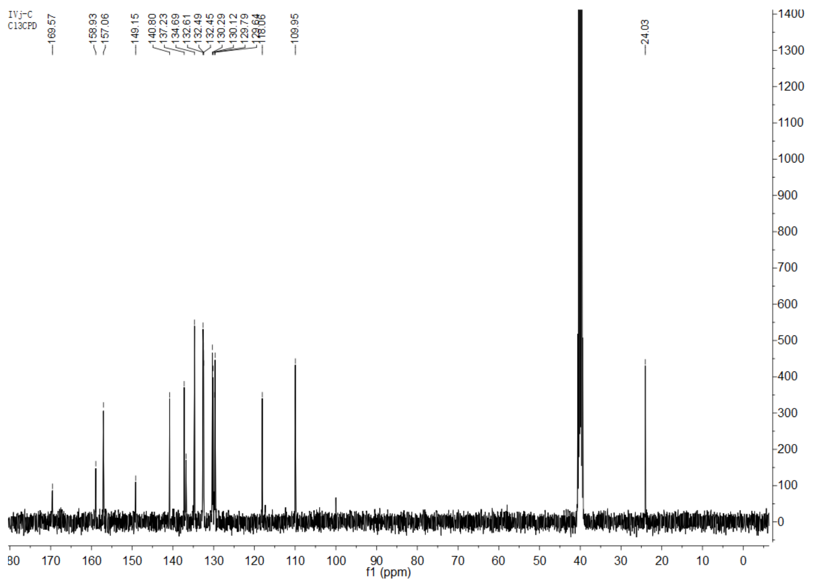

^{13}C NMR spectra for compound 9j.

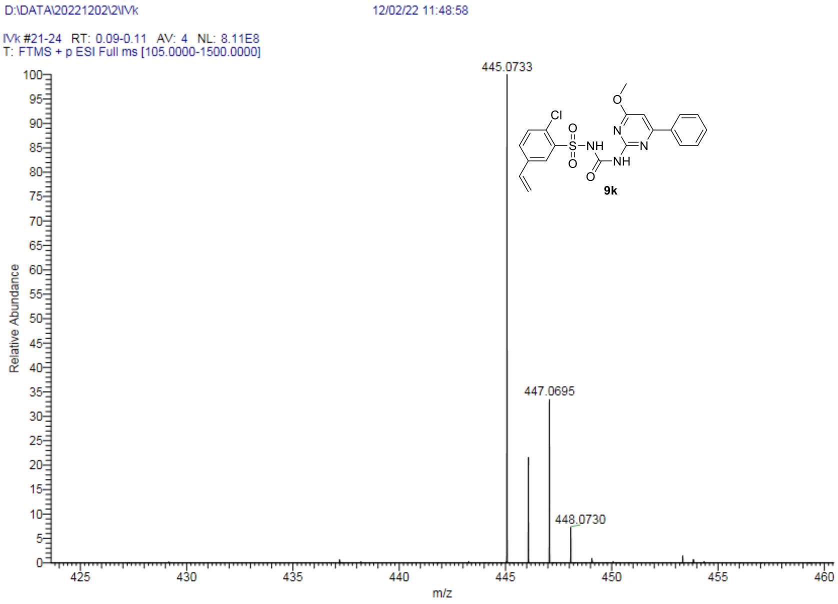

HRMS spectra for compound 9k.

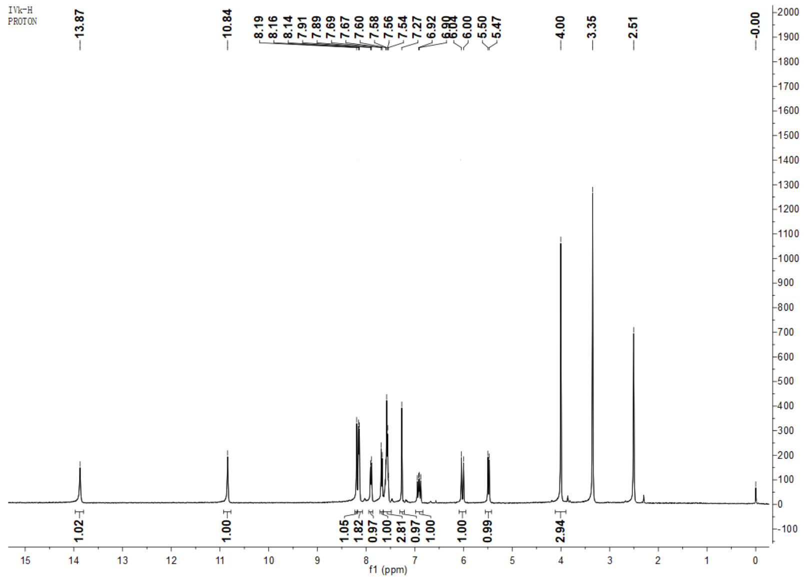

¹H NMR spectra for compound 9k.

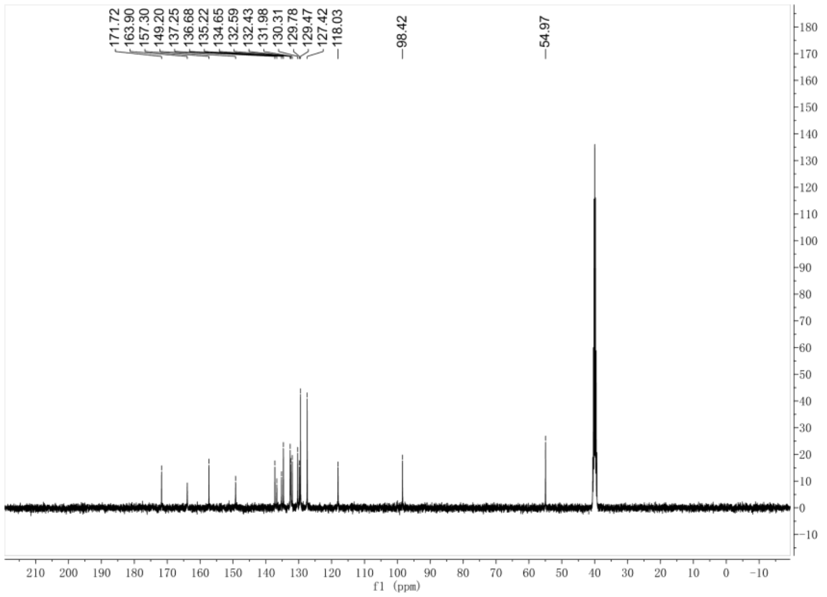

¹³C NMR spectra for compound 9k.

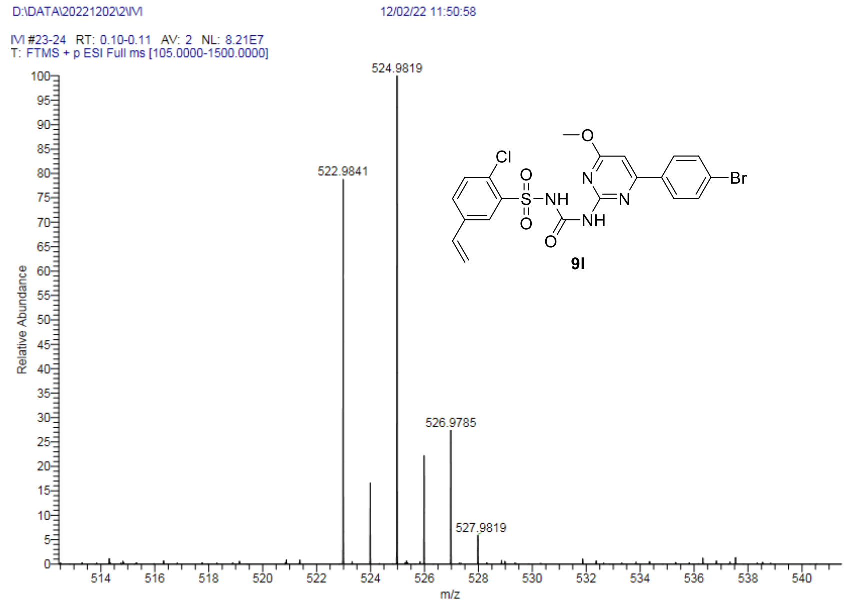

HRMS spectra for compound 9l.

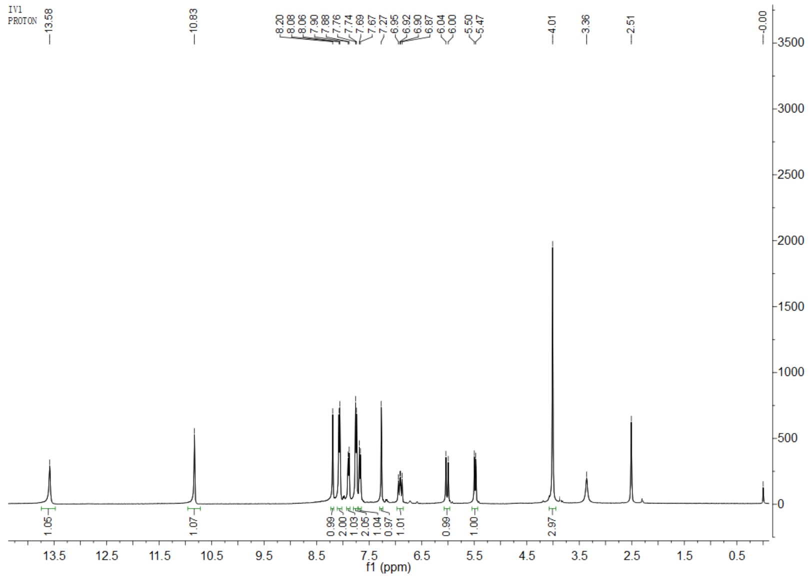

¹H NMR spectra for compound 9l.

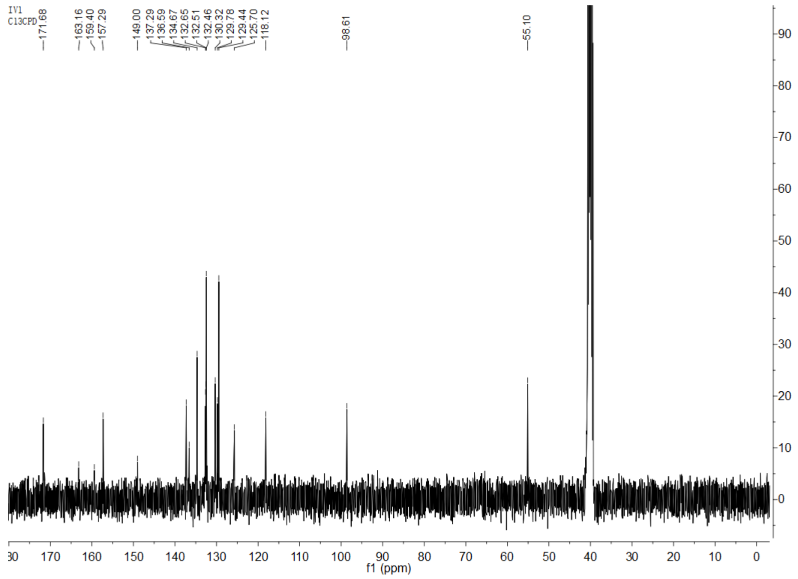

^{13}C spectra for compound 9l.

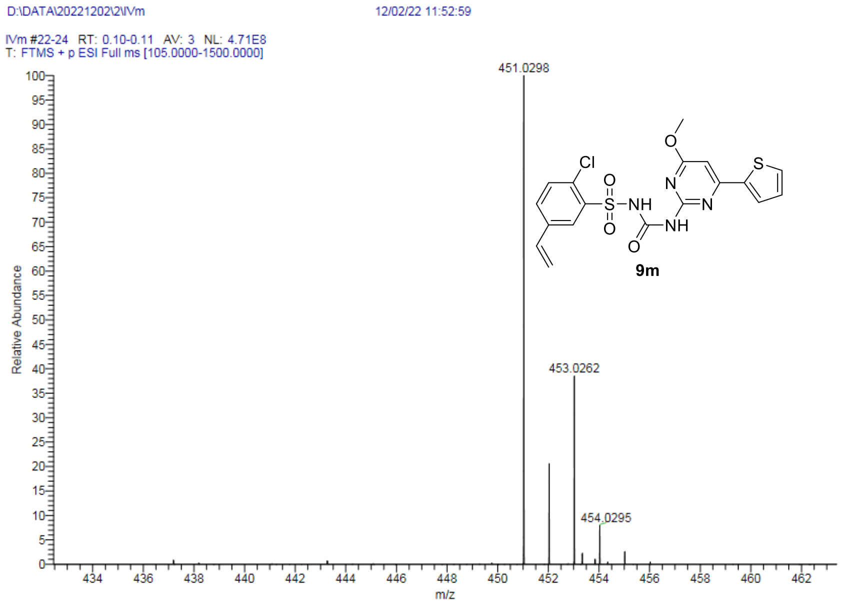

HRMS spectra for compound 9m.

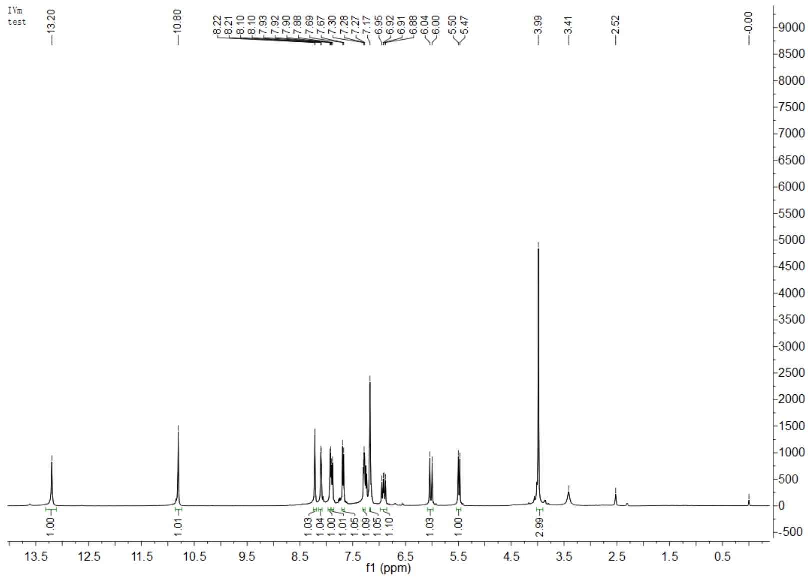

^{1}H NMR spectra for compound 9m.

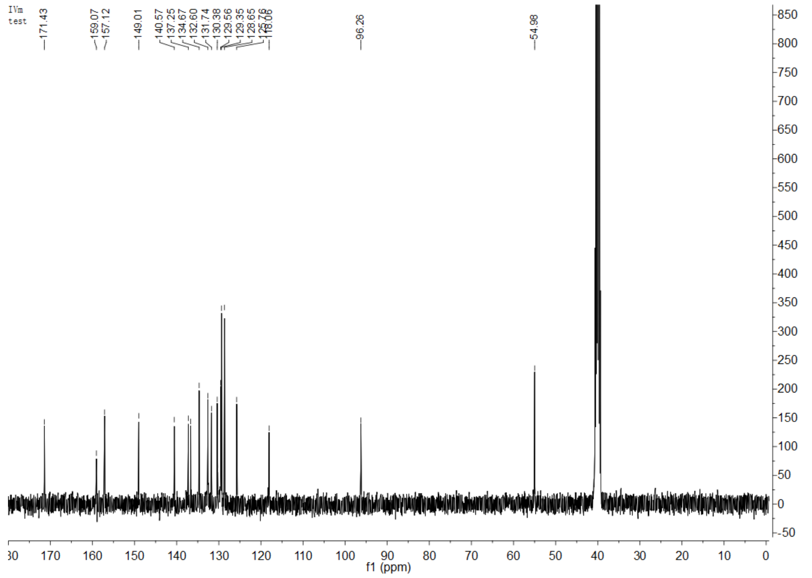

^{13}C spectra for compound 9m.

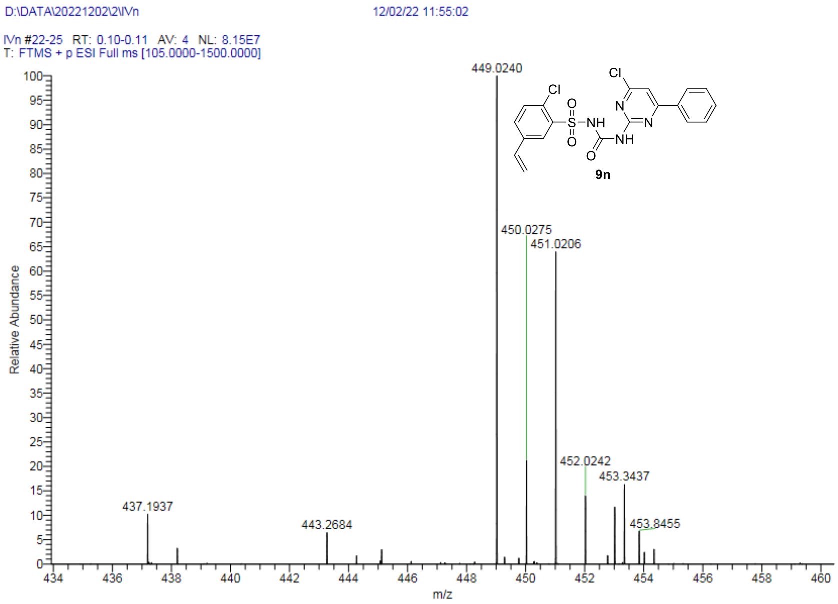

HRMS spectra for compound 9n.

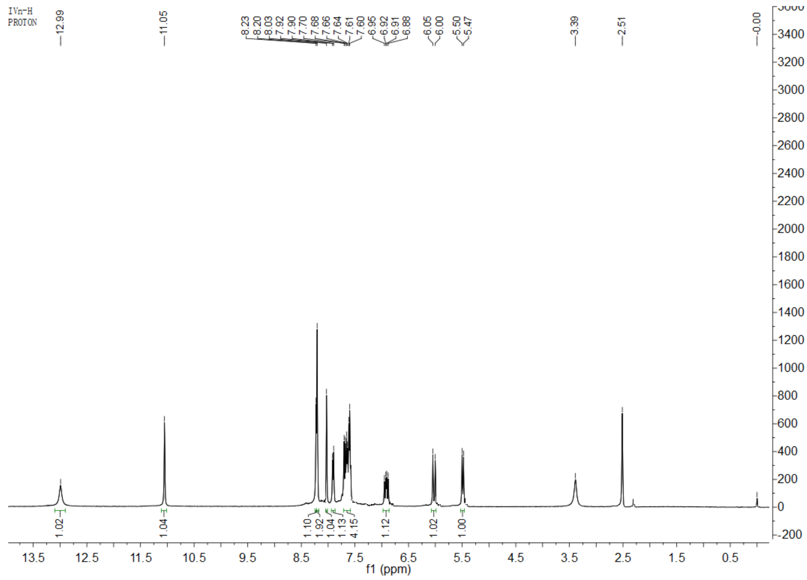

¹H NMR spectra for compound 9n.

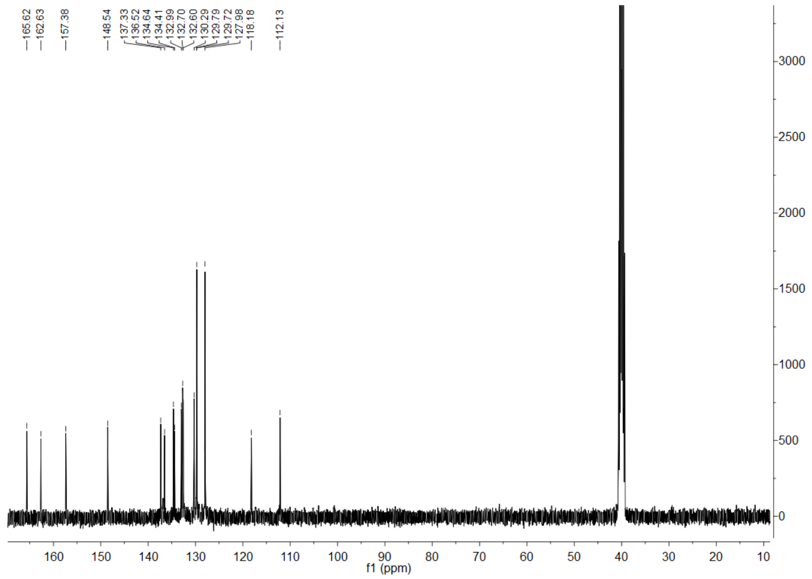

^{13}C spectra for compound 9n.

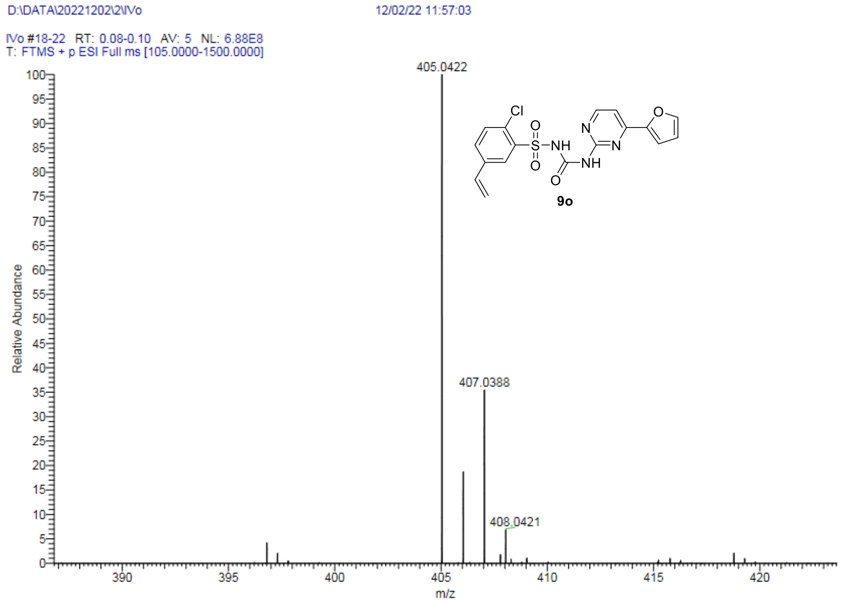

HRMS spectra for compound 9o.

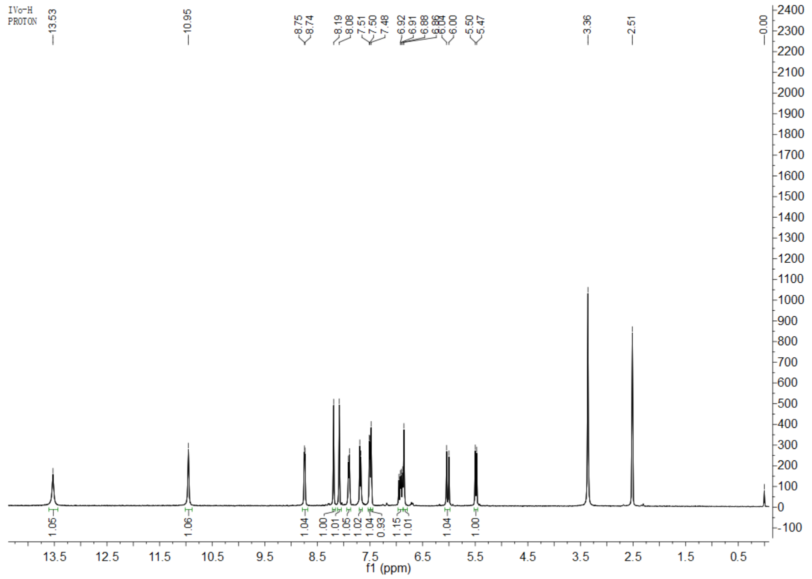

¹H NMR spectra for compound 9o.

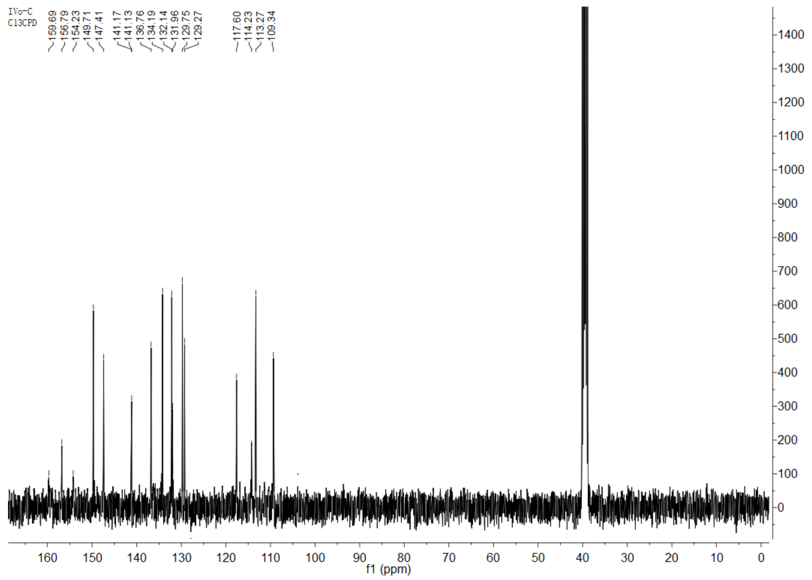

¹³C spectra for compound 9o.

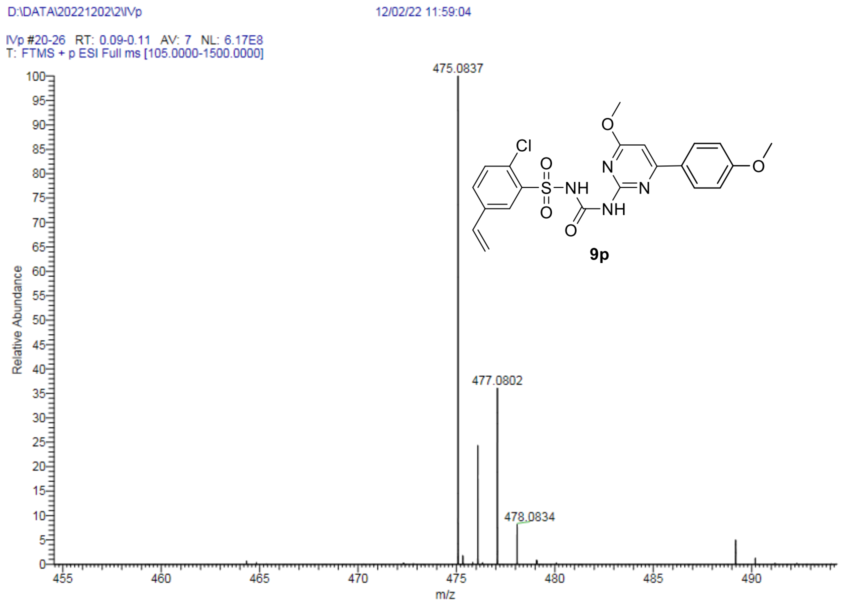

HRMS spectra for compound 9p.

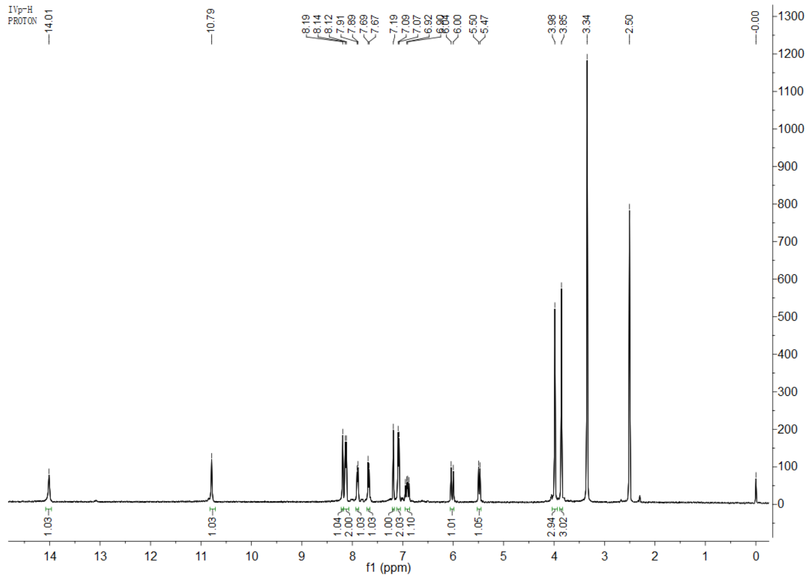

^{1}H NMR spectra for compound 9p.

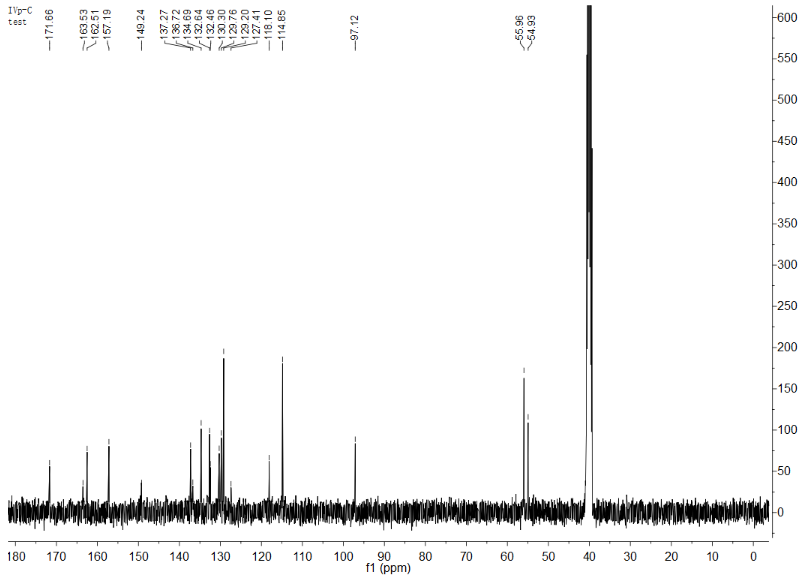

^{13}C spectra for compound 9p.

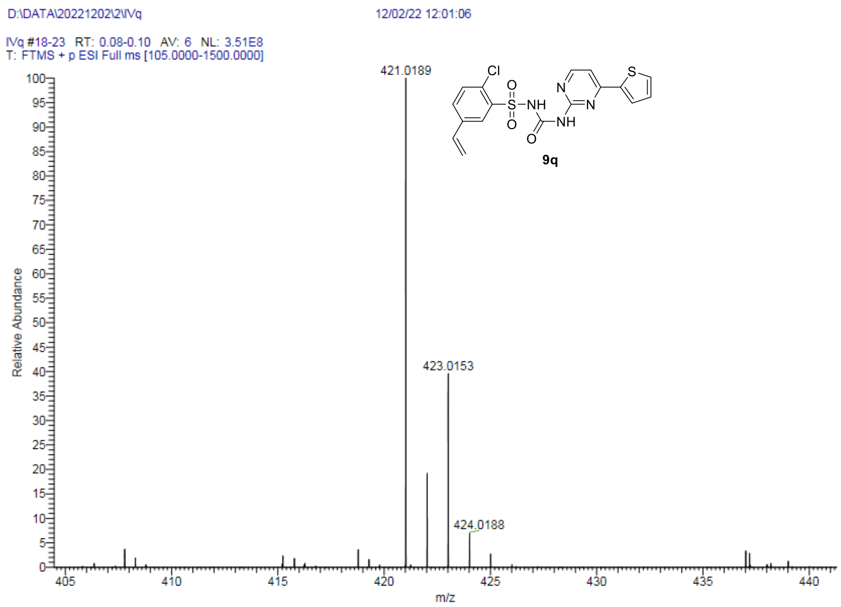

HRMS spectra for compound 9q.

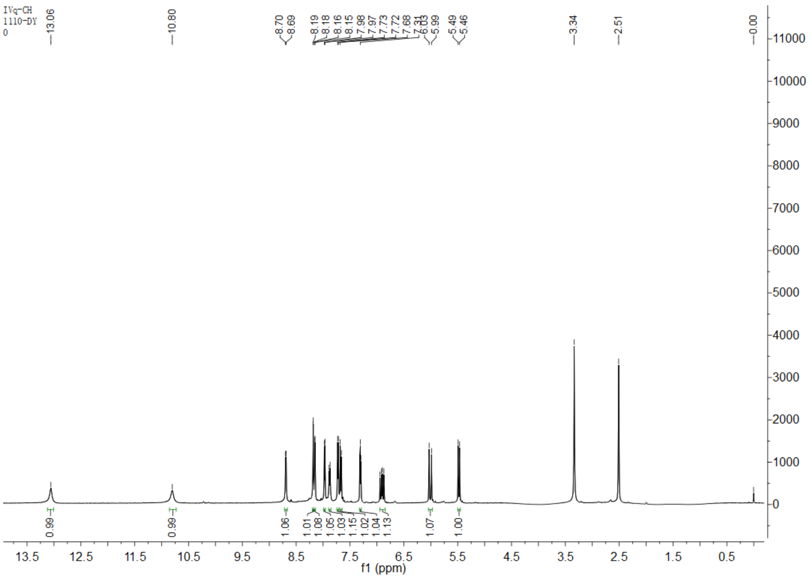

¹H NMR spectra for compound 9q.

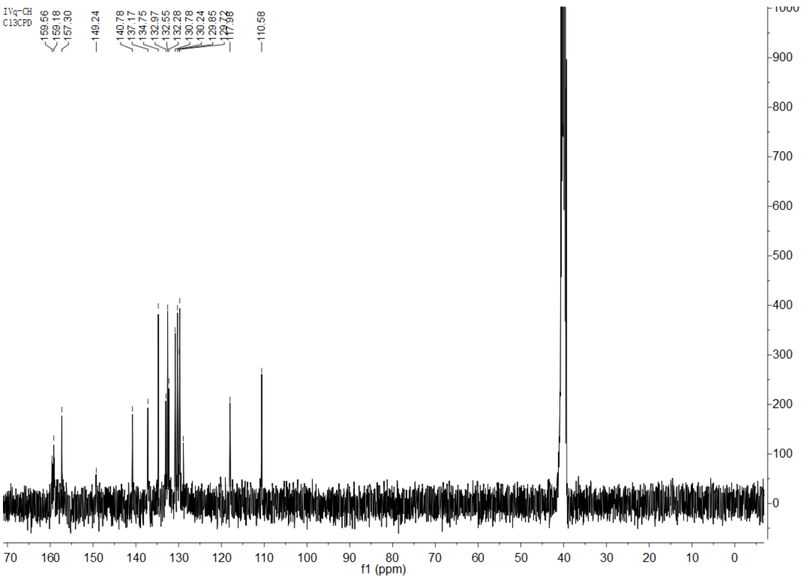

¹³C spectra for compound 9q.

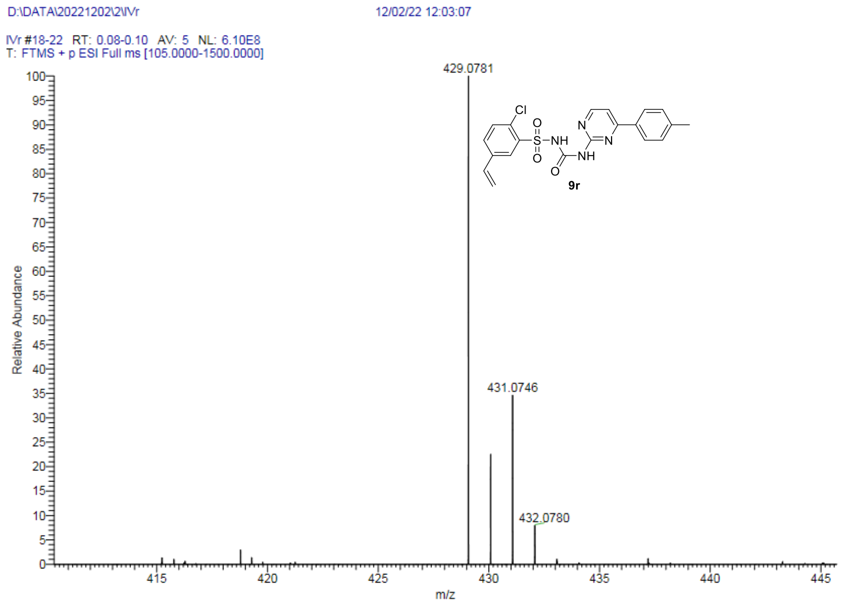

HRMS spectra for compound 9r.

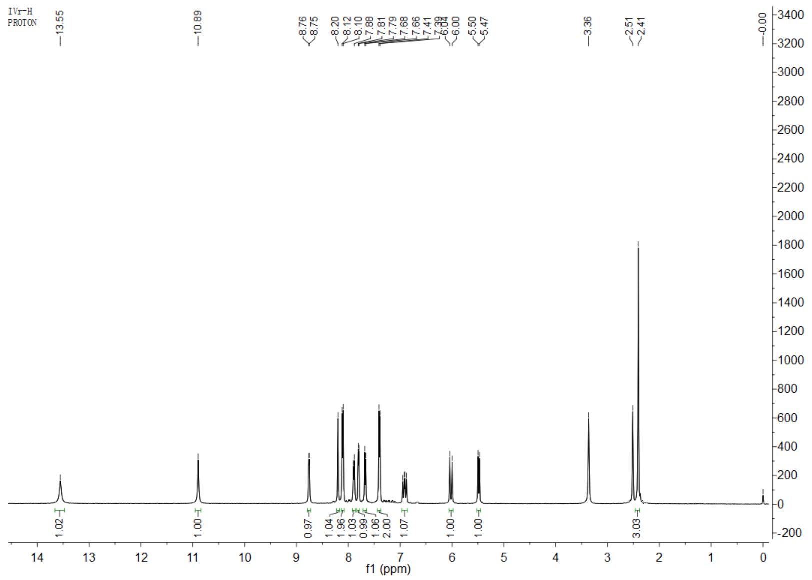

^{1}H NMR spectra for compound 9r.

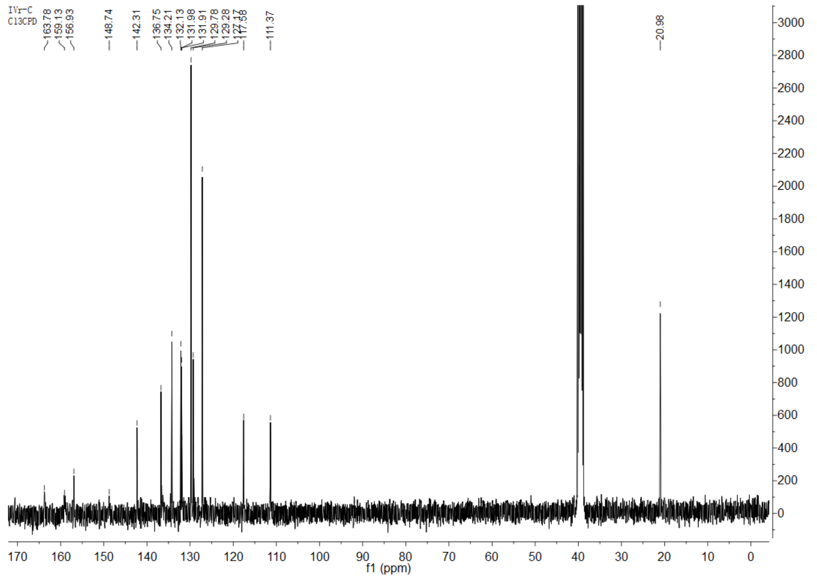

^{13}C spectra for compound 9r.

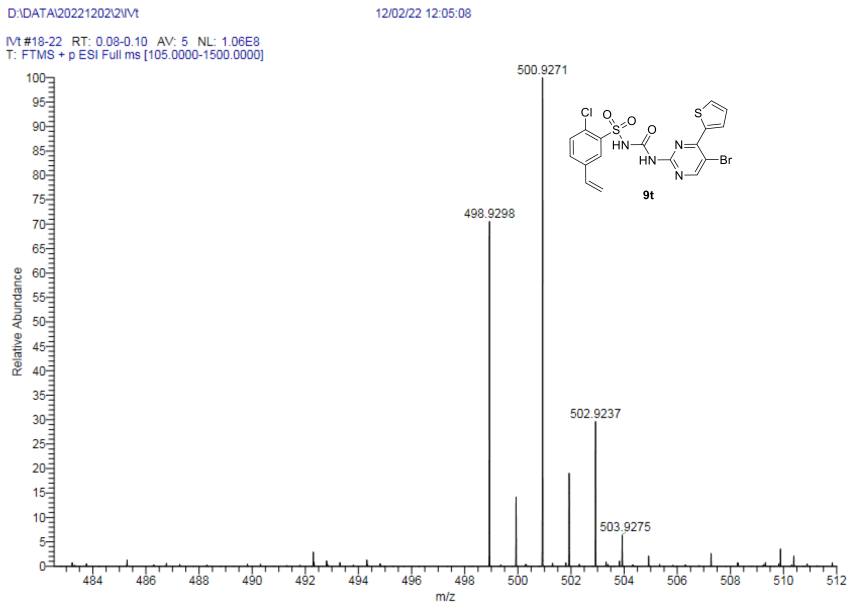

HRMS spectra for compound 9t.

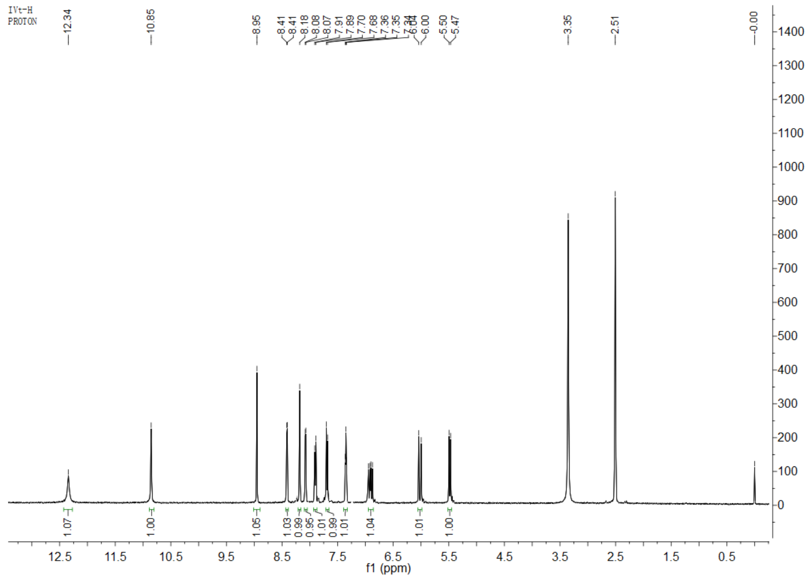

¹H NMR spectra for compound 9t.

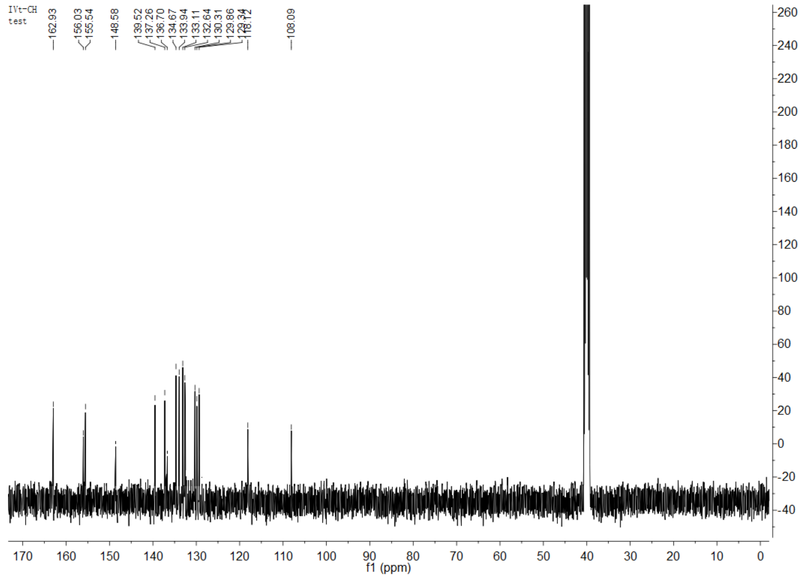

¹³C spectra for compound 9t.

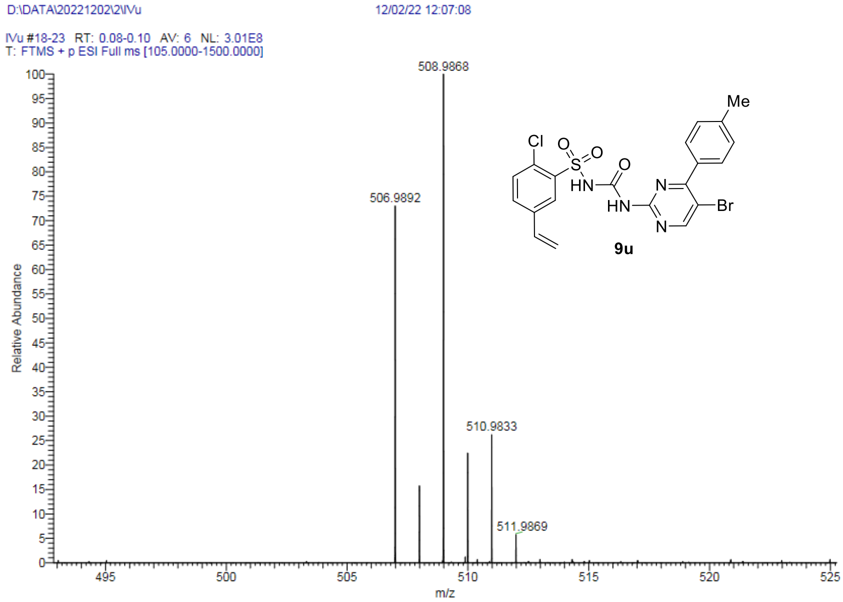

HRMS spectra for compound 9u.

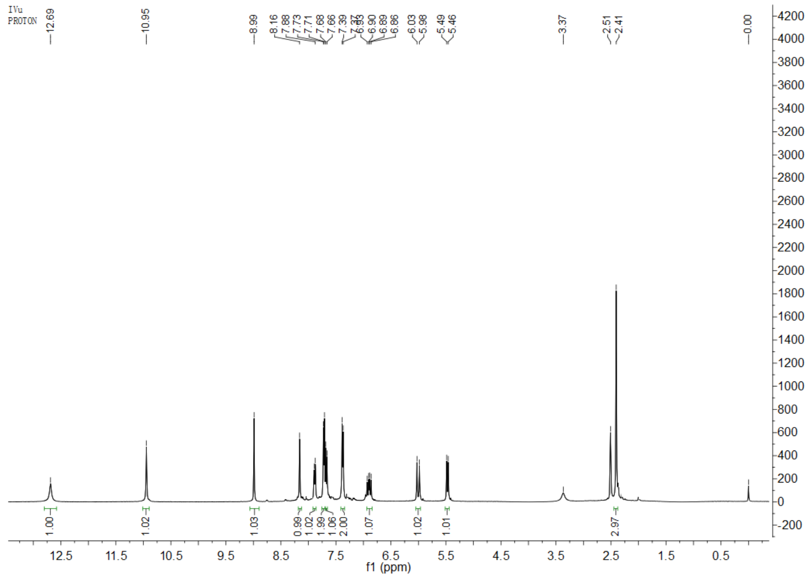

¹H NMR spectra for compound 9u.

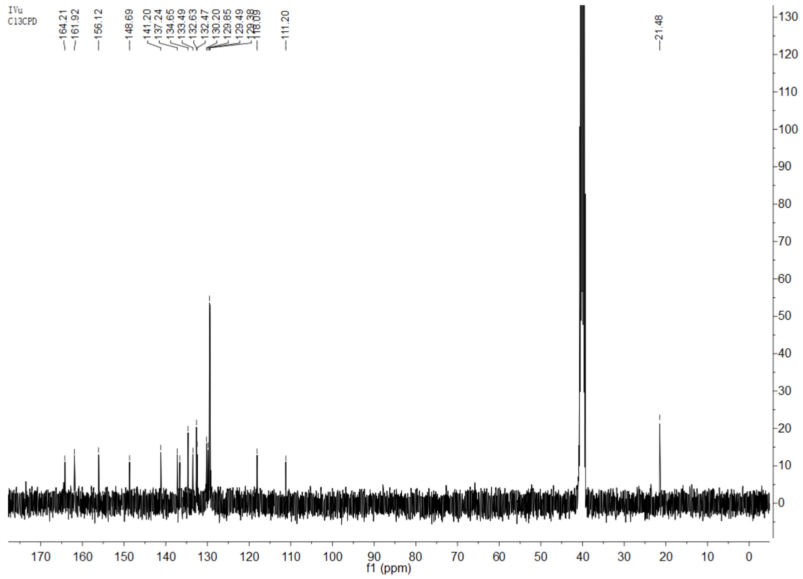

^{13}C spectra for compound 9u.
